# Synthesis and Biochemical Evaluation of Baicalein Prodrugs

**DOI:** 10.3390/pharmaceutics13091516

**Published:** 2021-09-19

**Authors:** Sang-Hyun Son, Jinhong Kang, Myunghwan Ahn, Soyeon Nam, Yong Woo Jung, Ki Yong Lee, Young Ho Jeon, Youngjoo Byun, Kiho Lee

**Affiliations:** 1 College of Pharmacy, Korea University, 2511 Sejong-ro, Sejong 30019, Korea; shson@korea.ac.kr (S.-H.S.); jhkang9361@gmail.com (J.K.); formnum@korea.ac.kr (M.A.); nsy7809@gmail.com (S.N.); yjung@korea.ac.kr (Y.W.J.); kylee11@korea.ac.kr (K.Y.L.); yhjeon@korea.ac.kr (Y.H.J.); 2 Institute of Pharmaceutical Science and Translational Research, Korea University, 2511 Sejong-ro, Sejong 30019, Korea

**Keywords:** baicalein, prodrug, carbamate, pharmacokinetics

## Abstract

Baicalein (5,6,7-trihydroxy-2-phenyl-4H-1-benzopyran-4-one), a flavonoid analog from *Scutellaria baicalensis*, possesses several pharmacological activities including antioxidant, antiproliferative, and anti-inflammatory activities. We previously reported that baicalein inhibits the thymic stromal lymphopoietin (TSLP)/TSLP receptor (TSLPR) signaling pathways and can be used as an active ingredient in the treatment of asthma and atopic dermatitis. However, baicalein is rapidly metabolized to baicalin and baicalein-6-*O*-glucuronide in vivo, which limits its preclinical and clinical use. In this study, we designed, synthesized, and evaluated baicalein prodrugs that protect the OH group at the 7-position of the A ring in baicalein with the amino acid carbamate functional group. Comprehensive in vitro and in vivo studies identified compound **2** as a baicalein prodrug candidate that improved the plasma exposure of baicalein in mouse animal studies. Our results demonstrated that this prodrug approach could be further adopted to discover oral baicalein prodrugs.

## 1. Introduction

**Figure 1:**
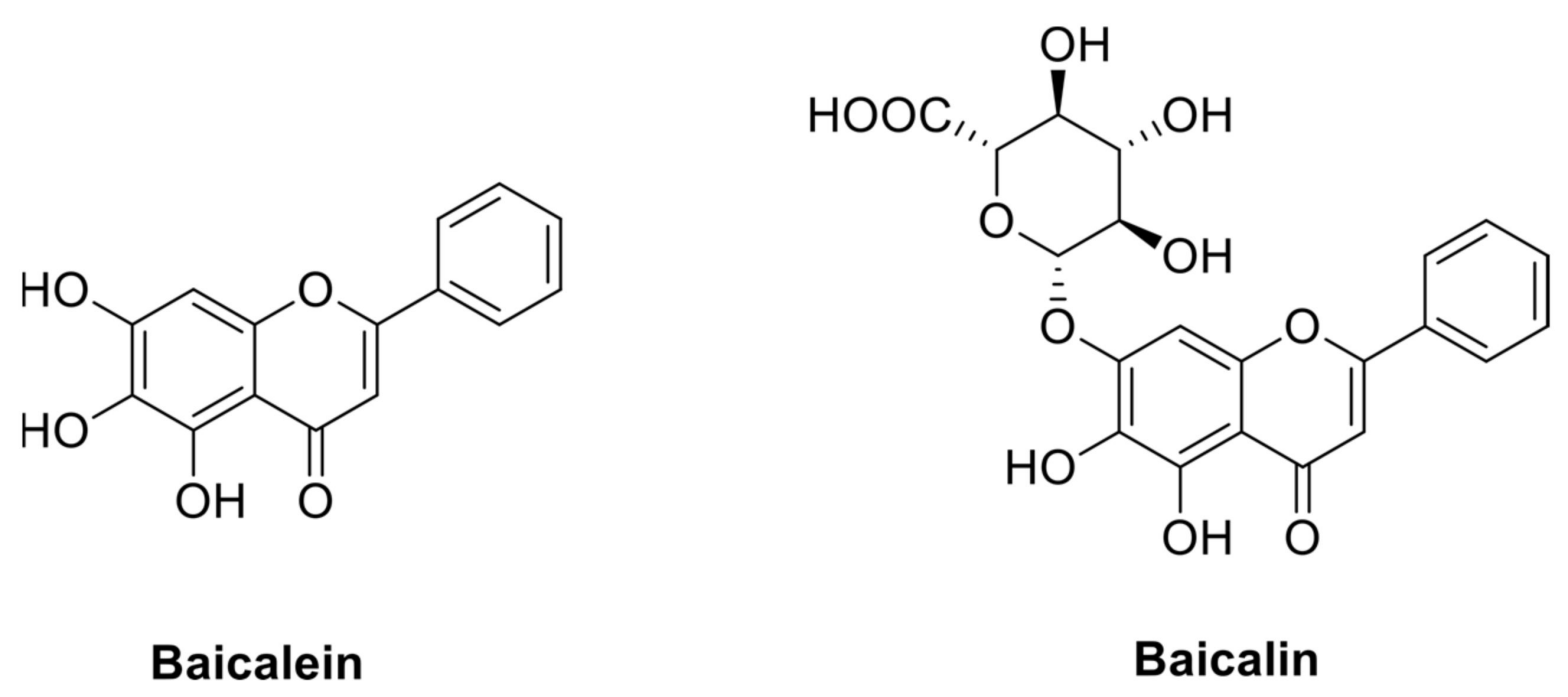
Chemical structure of baicalein and baicalin.

Baicalein (5,6,7-trihydroxy-2-phenyl-4H-1-benzopyran-4-one; Figure 1) is a flavonoid and a major component of *Scutellaria baicalensis* (*S. baicalensis*). It possesses various beneficial pharmacological activities, including anti-oxidative, anti-proliferative, anti-platelet aggregation, anti-thrombotic, reactive oxygen species (ROS)-scavenging, and anti-angiogenic activities [1,2,3,4,5]. Baicalein also inhibits the thymic stromal lymphopoietin (TSLP)/TSLP receptor (TSLPR) signaling pathways and may be utilized as an agent to treat asthma and atopic dermatitis [6]. However, oral and intravenous administration of baicalein from in vivo studies showed that the systemic bioavailability of baicalein was very low [7]. The phase II metabolism of baicalein occurs rapidly during the first-pass in the liver [7]. Baicalein is mainly metabolized to baicalin and baicalein-6-*O*-glucuronide by uridine diphosphate (UDP)-glucuronosyltransferase in vivo. Baicalin (Figure 1), along with baicalein-7-*O*-glucuronide, is also a key component of *S. baicalensis* that exhibits anti-inflammatory and anti-tumor activities [8,9]. Baicalin is metabolized in vivo to baicalein by the β-glucuronidase [10].

The prodrug approach is a chemical modification strategy that aims to optimize pharmacokinetic properties by modulating physicochemical properties including solubility, biochemical metabolism, and toxicity. A prodrug is an inactive compound which is transformed via chemical or enzymatic cleavage into the active molecule. As the prodrug approach generally offers chemical stability, it can be stable in the gastrointestinal tract and still be biotransformed by CYP450 in the liver and esterase in the blood.

Amino acid carbamates have recently been reported as resveratrol prodrugs [11]. Resveratrol, like many other polyphenolic natural products, such as baicalein, is also known to be rapidly metabolized by phase II biotransformation reactions, thereby showing an extensive first-pass effect and poor oral absorption [11]. Natural amino acids consisting of a pro-moiety with several advantages, such as increased water solubility, carrier-mediated transport, and safety [12,13], were coupled to the phenol groups of resveratrol via carbamate moieties to protect from extensive phase II metabolism [11]. This study, although only partially successful with suboptimal bioconversion, demonstrated the feasibility of the amino acid carbamate prodrug approach for improving the oral delivery of phenolic compounds [11].

Herein, we report the design and synthesis of amino acid carbamate prodrugs of baicalein and their physicochemical properties, metabolic stability, bioconversion, and pharmacokinetic characteristics. The introduction of the carbamate functional group at the 7-position of baicalein was successfully achieved in three synthetic steps. Our results demonstrate that this prodrug approach improved the oral absorption of baicalein and may be further adopted to develop oral baicalein prodrugs.

## 2. Materials and Methods

### 2.1. General

All the chemicals and solvents used in the organic synthesis were purchased from Sigma-Aldrich, TCI, or Acros organics and were used without further purification. Baicalein, trifluoroacetic acid (TFA), glipizide, 7-EC, enalapril maleate, formic acid, MeOH, 1-octanol, UDPGA, NADPH, PAPS, GSH, hydroxypropyl-β-cyclodextrin (HPβCD), MgCl_2_, and alamethicin were purchased from Sigma-Aldrich. Tris-hydrochloride (Tris-HCl) was purchased from Roche Diagnostics Gmb. HPLC-grade water and acetonitrile were purchased from Avantor Performance Materials. Pooled mouse liver S9 fractions were purchased from BD Biosciences. Reactions were monitored by TLC on 0.25 mm Merck precoated silica gel plates (60 F_254_). Reaction progress was monitored by TLC analysis using a UV lamp and/or KMnO_4_ staining for detection purposes. Column chromatography was performed on silica gel (230–400 mesh, Merck, Darmstadt, Germany). ^1^H and ^13^C NMR spectra were recorded at room temperature (298 K) in CDCl_3_ (7.26/77.16 ppm) or DMSO-d^6^ (2.50/39.50 ppm) on Bruker Ultrashield 600 MHz Plus spectrometer and referenced to an internal solvent. Chemical shifts are reported in parts per million (ppm). Coupling constants (*J*) are given in Hertz. Splitting patterns are indicated as s, singlet; d, doublet; t, triplet; q, quartet; m, multiplet; br, broad for ^1^H NMR data. High resolution mass spectra (HRMS) were recorded on an Agilent 6530 Accurate mass Q-TOF LC/MS spectrometer. Low resolution mass spectra (LRMS) analyses were obtained from an API 150EX ESI-MS spectrometer. The purity of final compounds was measured by analytical reverse-phase HPLC on an Agilent 1260 Infinity (Agilent) with a C18 column (Phenomenex, 150 mm × 4.6 mm, 3 μm, 110 Å).

### 2.2. General Procedure for the Preparation of Activated 4-Nitrophenyl Urethanes (***1b**–**7b***)

*N*,*N*-Diisopropylethylamine (DIPEA, 2.86 mL, 16.4 mmol, 2.0 eq.) was added to a stirred a solution of amino acid *tert*-butyl ester **1a–7a** (8.2 mmol, 1.0 eq.) and bis(4-nitrophenyl) carbonate (2.74 g, 9.0 mmol, 1.1 eq.) in THF (15 mL) at 0 °C, and stirring was continued for 12 h at room temperature. The reaction mixture was evaporated, and the residue was purified by silica gel column chromatography eluted with dichloromethane (DCM)/MeOH (100:1 to 50:1, *v*/*v*) to give 4-nitrophenyl urethanes **1b–7b**.

*(S)-tert-Butyl 4-methyl-2-(((4-nitrophenoxy)carbonyl)amino)pentanoate* (**1b**). Pale yellow oil, 52% yield. ^1^H NMR (600 MHz, CDCl_3_) δ 8.25 (d, *J* = 9.0 Hz, 2H), 7.34 (d, *J* = 9.0 Hz, 2H), 5.68 (d, *J* = 9.0 Hz, 1H), 4.24 (q, *J* = 4.8 Hz, 1H), 1.85–1.52 (m, 2H), 1.51 (s, 9H), 1.49 (s, 1H), 1.03 (d, *J* = 6.6 Hz, 3H), 0.96 (d, *J* = 6.6 Hz, 3H). LRMS (ESI) *m/z* 353.2 [M+H]^+^.

*(S)-tert-Butyl 3-methyl-2-(((4-nitrophenoxy)carbonyl)amino)butanoate* (**2b**). Pale yellow oil, 67% yield. ^1^H NMR (600 MHz, CDCl_3_) δ 8.24 (d, *J* = 9.0 Hz, 2H), 7.33 (d, *J* = 9.0 Hz, 2H), 5.56 (d, *J* = 9.0 Hz, 1H), 4.24 (q, *J* = 4.8 Hz, 1H), 2.28–2.21 (m, 1H), 1.51 (s, 9H), 1.03 (d, *J* = 6.6 Hz, 3H), 0.96 (d, *J* = 6.6 Hz, 3H); ^13^C NMR (150 MHz, CDCl_3_) δ 170.7, 155.9, 153.2, 144.6, 124.9, 121.6, 82.3, 67.8, 59.6, 31.3, 27.7, 25.5, 18.8, 17.3. LRMS (ESI) *m/z* 339.2 [M+H]^+^.

*(2S,3R)-tert-Butyl 3-methyl-2-(((4-nitrophenoxy)carbonyl)amino)pentanoate* (**3b**). Pale yellow oil, 64% yield. ^1^H NMR (600 MHz, CDCl_3_) δ 8.24 (d, *J* = 9.6 Hz, 2H), 7.33 (d, *J* = 9.6 Hz, 2H), 5.70 (d, *J* = 8.4 Hz, 1H), 4.27 (q, *J* = 4.8 Hz, 1H), 2.05–1.87 (m, 1H), 1.51 (s, 9H), 1.51–1.47 (m, 1H), 1.35–1.16 (m, 1H), 0.99 (d, *J* = 7.2 Hz, 3H), 0.97 (d, *J* = 7.2 Hz, 3H). LRMS (ESI) *m/z* 353.2 [M+H]^+^.

*(2S,3S)-tert-Butyl 3-(tert-butoxy)-2-(((4-nitrophenoxy)carbonyl)amino)butanoate* (**4b**). Pale yellow oil, 56% yield. ^1^H NMR (600 MHz, CDCl_3_) δ 8.24 (d, *J* = 5.4 Hz, 2H), 7.33 (d, *J* = 5.4 Hz, 2H), 5.88 (d, *J* = 9.0 Hz, 1H), 4.27 (q, *J* = 1.8 Hz, 1H), 4.11 (d, *J* = 1.8 Hz, 1H), 1.50 (s, 9H), 1.27 (d, *J* = 6.6 Hz, 3H), 1.19 (s, 9H). LRMS (ESI) *m/z* 341.1 [M+H]^+^.

*(S)-tert-Butyl 2-(((4-nitrophenoxy)carbonyl)amino)-3-phenylpropanoate* (**5b**). Pale yellow oil, 61% yield. ^1^H NMR (600 MHz, CDCl_3_) δ 8.24 (d, *J* = 7.2 Hz, 2H), 7.36–7.31 (m, 2H), 7.29–7.26 (m, 3H), 7.23–7.19 (m, 2H), 5.62 (d, *J* = 7.8 Hz, 1H), 4.27 (q, *J* = 2.4 Hz, 1H), 3.31–3.08 (m, 2H), 1.45 (s, 9H). LRMS (ESI) *m/z* 387.2 [M+H]^+^.

*(S)-tert-Butyl 2-(((4-nitrophenoxy)carbonyl)amino)propanoate* (**6b**). Pale yellow oil, 63% yield. ^1^H NMR (600 MHz, CDCl_3_) δ 8.23 (d, *J* = 9.0 Hz, 2H), 7.32 (d, *J* = 9.0 Hz, 2H), 5.86 (d, *J* = 7.2 Hz, 1H), 4.33–4.29 (m, 1H), 1.49(s, 9H), 1.47 (d, *J* = 7.2 Hz, 1H); ^13^C NMR (150 MHz, CDCl_3_) δ 171.7, 155.6, 152.4, 144.9, 125.2, 122.1, 82.7, 50.5, 28.0, 18.7. LRMS (ESI) *m/z* 311.1 [M+H]^+^.

*tert-Butyl 2-(((4-nitrophenoxy)carbonyl)amino)acetate* (**7b**). Pale yellow oil, 60% yield. ^1^H NMR (600 MHz, CDCl_3_) δ 8.19 (d, *J* = 10.2 Hz, 2H), 7.33 (d, *J* = 10.2 Hz, 2H), 6.26 (t, *J* = 6.0 Hz, 1H), 3.95 (d, *J* = 5.4 Hz, 2H), 1.49 (s, 9H); ^13^C NMR (150 MHz, CDCl_3_) δ 168.5, 155.7, 153.2, 144.5, 124.8, 121.8, 82.3, 43.2, 27.7. LRMS (ESI) *m/z* 297.1 [M+H]^+^.

### 2.3. General Procedure for the Preparation of 7-N-Monosubstituted-Baicalein Carbamate Esters (***1c**–**7c***)

DIPEA (0.55 mL, 3.16 mmol, 2.0 eq.) was added to a stirred a solution of baicalein (1.58 mmol, 1.0 eq.) and activated 4-nitrophenyl urethane **1a–7a** (1.58 mmol, 1.0 eq.) in THF (10 mL) at 0 °C, and stirring was continued for 12 h at room temperature, when TLC (DCM/MeOH, 20/1, *v*/*v*) indicated that reaction was complete. The reaction mixture was evaporated, and the residue was purified twice by silica gel column chromatography (DCM/acetone, 100/1 to 50/1, *v*/*v*, followed by DCM/MeOH, 100/1, *v*/*v*) to provide 7-*N*-monosubstituted-baicalein carbamate esters **1c–7c**.

*(S)-tert-Butyl 2-((((5,6-dihydroxy-4-oxo-2-phenyl-4H-chromen-7-yl)oxy)carbonyl)amino)-4-methylpentanoate* (**1c**). Yellow powder, 78% yield. ^1^H NMR (600 MHz, DMSO-d^6^) δ 12.93 (s, 1H), 8.13–8.07 (m, 3H), 7.66–7.56 (m, 3H), 7.01 (s, 1H), 6.66 (s, 1H), 3.97–3.91 (m, 1H), 1.79–1.70 (m, 1H), 1.64–1.56 (m, 1H), 1.54–1.46 (m, 1H), 1.40 (s, 9H), 0.93 (d, *J* = 6.6 Hz, 3H), 0.89 (d, *J* = 6.6 Hz, 3H); ^13^C NMR (150 MHz, DMSO-d^6^) δ 182.7, 172.1, 163.9, 158.1, 154.2, 153.9, 153.5, 132.6, 131.2, 129.6, 127.0, 123.4, 105.3, 104.4, 94.5, 81.0, 53.8, 28.1, 24.7, 23.2, 21.9. HRMS (ESI) *m/z* calculated for C_26_H_29_NO_8_^–^ [M-H]^–^: 482.1823; found: 428.1828.

*(S)-tert-Butyl 2-((((5,6-dihydroxy-4-oxo-2-phenyl-4H-chromen-7-yl)oxy)carbonyl)amino)-3-methylbutanoate* (**2c**). Yellow powder, 47% yield. ^1^H NMR (600 MHz, DMSO-d^6^) δ 12.94 (s, 1H), 8.10–8.06 (m, 2H), 8.04 (d, *J* = 8.4 Hz, 1H), 7.63–7.56 (m, 3H), 6.99 (s, 1H), 6.66 (s, 1H), 3.83 (q, *J* = 6.6 Hz, 1H), 2.10–2.04 (m, 1H), 1.44 (s, 9H), 0.96 (d, *J* = 5.4 Hz, 3H), 0.94 (d, *J* = 5.4 Hz, 3H); ^13^C NMR (150 MHz, DMSO-d^6^) δ 182.2, 170.5, 163.5, 157.6, 153.8, 153.7, 153.0, 132.1, 130.7, 129.2, 126.5, 122.9, 104.8, 104.0, 94.1, 80.6, 60.6, 54.9, 30.1, 27.7, 18.9, 18.1. HRMS (ESI) *m/z* calculated for C_25_H_27_NO_8_^–^ [M-H]^–^: 468.1664; found: 468.1645.

*(2S,3R)-tert-Butyl 2-((((5,6-dihydroxy-4-oxo-2-phenyl-4H-chromen-7-yl)oxy)carbonyl)amino)-3-methylpentanoate* (**3c**). Yellow powder, 69% yield. ^1^H NMR (600 MHz, DMSO-d^6^) δ 12.95 (s, 1H), 8.09 (d, *J* = 7.2 Hz, 2H), 8.06 (d, *J* = 8.4 Hz, 1H), 7.63–7.56 (m, 3H), 7.01 (s, 1H), 6.66 (s, 1H), 3.88 (q, *J* = 6.6 Hz, 1H), 1.89–1.73 (m, 1H), 1.44 (s, 9H), 1.38–1.21 (m, 2H), 0.92 (d, *J* = 7.2 Hz, 3H), 0.89 (d, *J* = 7.2 Hz, 3H); ^13^C NMR (150 MHz, DMSO-d^6^) δ 182.2, 170.5, 163.5, 157.6, 153.8, 153.7, 153.7, 153.0, 132.1, 130.7, 129.2, 126.5, 122.9, 104.8, 104.0, 94.1, 80.6, 59.5, 36.6, 27.7, 24.9, 15.4, 11.3. HRMS (ESI) *m/z* calculated for C_26_H_29_NO_8_^–^ [M-H]^–^: 482.1820; found: 482.1798.

*(2S,3R)-tert-Butyl 3-(tert-butoxy)-2-((((5,6-dihydroxy-4-oxo-2-phenyl-4H-chromen-7-yl)oxy)carbonyl)amino)butanoate* (**4c**). Yellow powder, 60% yield. ^1^H NMR (600 MHz, DMSO-d^6^) δ 12.94 (s, 1H), 8.07 (d, *J* = 7.2 Hz, 2H), 7.64–7.53 (m, 3H), 7.44 (d, *J* = 9.0 Hz, 1H), 6.99 (s, 1H), 6.64 (s, 1H), 4.06–4.00 (m, 1H), 3.97 (dd, *J* = 4.0 and 9.3 Hz, 1H), 1.42 (s, 9H), 1.14 (s, 9H), 1.13 (d, *J* = 6.0 Hz, 3H); ^13^C NMR (150 MHz, DMSO-d^6^) δ 182.2, 169.3, 163.5, 157.6, 153.8, 153.6, 153.0, 132.1, 130.7, 129.1, 126.5, 122.9, 104.8, 104.0, 94.1, 80.9, 73.4, 67.2, 60.5, 28.3, 27.7, 19.9. HRMS (ESI) *m/z* calculated for C_28_H_33_NO_9_^–^ [M-H]^–^: 526.2082; found: 526.2099.

(*S)-tert-Butyl 2-((((5,6-dihydroxy-4-oxo-2-phenyl-4H-chromen-7-yl)oxy)carbonyl)amino)-3-phenylpropanoate* (**5c**). Yellow powder, 66% yield. ^1^H NMR (600 MHz, DMSO-d^6^) δ 8.21 (d, *J* = 7.8 Hz, 1H), 8.08 (d, *J* = 7.2 Hz, 1H), 7.63–7.54 (m, 3H), 7.32–7.21 (m, 5H), 6.99 (s, 1H), 6.63 (s, 1H), 4.13 (q, *J* = 8.4 Hz, 1H), 3.03–2.95 (m, 2H), 1.34 (s, 9H); ^13^C NMR (150 MHz, DMSO-d^6^) δ 182.2, 170.5, 163.4, 153.8, 153.3, 153.0, 137.3, 132.1, 130.7, 129.4, 129.3, 129.2, 128.2, 126.5, 126.5, 122.9, 104.8, 103.8, 94.1, 80.7, 56.5, 36.8, 27.5. HRMS (ESI) *m/z* calculated for C_29_H_27_NO_8_– [M-H]^–^: 516.1659; found: 516.1673.

*(S)-tert-Butyl 2-((((5,6-dihydroxy-4-oxo-2-phenyl-4H-chromen-7-yl)oxy)carbonyl)amino)propanoate* (**6c**). Yellow powder, 80% yield. ^1^H NMR (600 MHz, DMSO-d^6^) δ 12.93 (s, 1H), 8.14 (d, *J* = 7.2 Hz, 1H), 8.09 (d, *J* = 7.2 Hz, 1H), 7.65–7.56 (m, 3H), 7.01 (s, 1H), 6.66 (s, 1H), 3.99–3.93 (m, 1H), 1.42 (s, 9H), 1.31 (d, *J* = 7.2 Hz, 3H); ^13^C NMR (150 MHz, DMSO-d^6^) δ 182.2, 172.0, 163.5, 157.6, 153.7, 153.1, 153.0, 132.1, 130.7, 129.2, 126.5, 122.8, 104.8, 104.0, 94.0, 80.4, 50.3, 27.6, 17.1. HRMS (ESI) *m/z* calculated for C_23_H_23_NO_8_^+^ [M+H]^+^: 442.1497; found: 442.1507.

*tert-Butyl 2-((((5,6-dihydroxy-4-oxo-2-phenyl-4H-chromen-7-yl)oxy)carbonyl)amino)acetate* (**7c**). Yellow powder, 74% yield. ^1^H NMR (600 MHz, DMSO-d^6^) δ 12.94 (s, 1H), 8.13–8.05 (m, 3H), 7.66–7.55 (m, 3H), 7.01 (s, 1H), 6.66 (s, 1H), 3.72 (d, *J* = 6.0 Hz, 2H), 1.43 (s, 9H); ^13^C NMR (150 MHz, DMSO-d^6^) δ 182.2, 168.9, 163.4, 157.5, 153.8, 153.7, 153.0, 132.1, 130.7, 129.1, 126.5, 122.8, 104.8, 103.9, 94.0, 80.7, 43.1, 27.7. HRMS (ESI) *m/z* calculated for C_22_H_21_NO_8_^+^ [M+Na]^+^: 450.1159; found: 450.1171.

### 2.4. General Procedure for the Tert-Butyl Ester Deprotection in Baicalein-Amino Acid Cabamoyl Conjugate (***1**–**7***)

The *tert*-butyl protected baicalein-amino acid carbamoyl conjugates **1c–7c** (0.20 mmol) was dissolved in DCM (3 mL) at 0 °C. Trifluoroacetic acid (TFA, 1 mL) was added and the reaction mixture was stirred for 6 h at room temperature, when TLC (DCM/MeOH, 20/1, *v*/*v*) indicated the disappearance of stating material. The solvent and residual TFA were removed under reduced pressure. The residue was suspended in DCM-hexane (24 mL, 1/5, *v*/*v*) and filtered, and the filtrate was washed with DCM-hexane (12 mL, 1/5, *v*/*v*) to yield the baicalein-amino acid carbamoyl conjugates (**1–7**).

*(S)-2-((((5,6-Dihydroxy-4-oxo-2-phenyl-4H-chromen-7-yl)oxy)carbonyl)amino)-4-methylpentanoic acid* (**1**). Yellow amorphous powder, quantitative yield. ^1^H NMR (600 MHz, DMSO-d^6^) δ 12.93 (s, 1H), 11.28 (s, 1H), 8.10 (d, *J* = 7.2 Hz, 1H), 8.08 (d, *J* = 10.8 Hz, 1H), 7.63 (t, *J* = 7.2 Hz, 1H), 7.59 (t, *J* = 7.2 Hz, 2H), 7.01 (s, 1H), 6.66 (s, 1H), 4.06–4.01 (m, 1H), 1.79–1.72 (m, 1H), 1.67–1.59 (m, 1H), 1.58–1.49 (m, 1H), 0.93 (d, *J* = 6.6 Hz, 3H), 0.90 (d, *J* = 6.6 Hz, 3H); ^13^C NMR (150 MHz, DMSO-d^6^) δ 182.2, 174.0, 163.5, 157.5, 153.7, 153.5, 153.0, 132.1, 130.7, 129.2, 126.5, 123.0, 104.8, 104.0, 94.0, 52.5, 24.3, 22.9, 21.3. HRMS (ESI) *m/z* calculated for C_22_H_21_NO_8_^+^ [M+H]^+^: 428.1340; found: 428.1351.

*(S)-2-((((5,6-Dihydroxy-4-oxo-2-phenyl-4H-chromen-7-yl)oxy)carbonyl)amino)-3-methylbutanoic acid* (**2**). Yellow amorphous powder, quantitative yield. ^1^H NMR (600 MHz, DMSO-d^6^) δ 12.93 (s, 1H), 11.33 (s, 1H), 8.09 (d, *J* = 7.2 Hz, 2H), 8.02 (d, *J* = 8.4 Hz, 1H), 7.64–7.58 (m, 3H), 7.01 (s, 1H), 6.67 (s, 1H), 3.91 (dd, *J* = 6.0 and 9.0 Hz, 1H), 2.17–2.07 (m, 1H), 0.95 (d, *J* = 7.2 Hz, 3H), 0.95 (d, *J* = 7.2 Hz, 3H); ^13^C NMR (150 MHz, DMSO-d^6^) δ 182.2, 172.9, 163.5, 157.5, 153.8, 153.7, 153.0, 132.1, 130.7, 129.2, 126.5, 123.0, 104.8, 104.0, 94.1, 59.8, 30.0, 19.1, 18.0. HRMS (ESI) *m/z* calculated for C_21_H_19_NO_8_^+^ [M+H]^+^: 414.1184; found: 414.1194.

*(2S,3R)-2-((((5,6-Dihydroxy-4-oxo-2-phenyl-4H-chromen-7-yl)oxy)carbonyl)amino)-3-methylpentanoic acid* (**3**). Yellow amorphous powder, quantitative yield. ^1^H NMR (600 MHz, DMSO-d^6^) δ 12.93 (s, 1H), 8.08 (d, *J* = 7.2 Hz, 2H), 8.01 (d, *J* = 8.4 Hz, 1H), 7.62 (t, *J* = 7.2 Hz, 1H), 7.58 (t, *J* = 7.8 Hz, 2H), 7.00 (s, 1H), 6.66 (s, 1H), 3.95 (dd, *J* = 6.6 and 8.4 Hz, 1H), 1.89–1.81 (m, 1H), 1.53–1.42 (m, 1H), 1.31–1.21 (m, 1H), 0.92 (d, *J* = 6.6 Hz, 3H), 0.89 (d, *J* = 7.8 Hz, 3H); ^13^C NMR (150 MHz, DMSO-d^6^) δ 182.2, 172.9, 163.5, 157.7, 153.7, 153.7, 153.0, 132.1, 130.7, 129.2, 126.5, 123.0, 104.8, 104.0, 94.1, 59.0, 36.5, 24.6, 15.5, 11.3. HRMS (ESI) *m/z* calculated for C_22_H_21_NO_8_^+^ [M+H]^+^: 428.1340; found: 428.1349.

*(2S,3R)-2-((((5,6-Dihydroxy-4-oxo-2-phenyl-4H-chromen-7-yl)oxy)carbonyl)amino)-3-hydroxybutanoic acid* (**4**). Yellow amorphous powder, quantitative yield. ^1^H NMR (600 MHz, DMSO-d^6^) δ 12.95 (s, 1H), 8.09 (d, *J* = 7.8 Hz, 2H), 7.67–7.53 (m, 3H), 7.48 (d, *J* = 9.0 Hz, 1H), 7.01 (s, 1H), 6.66 (s, 1H), 4.19–4.11 (m, 1H), 4.09–3.99 (m, 1H), 1.16 (d, *J* = 6.0 Hz, 3H); ^13^C NMR (150 MHz, DMSO-d^6^) δ 182.2, 171.9, 163.5, 157.5, 153.8, 153.7, 153.0, 132.1, 130.7, 129.2, 126.5, 122.9, 104.9, 104.0, 94.1, 66.8, 60.2, 20.2. HRMS (ESI) *m/z* calculated for C_20_H_17_NO_9_^+^ [M+H]^+^: 416.0976; found: 416.0984.

*(S)-2-((((5,6-Dihydroxy-4-oxo-2-phenyl-4H-chromen-7-yl)oxy)carbonyl)amino)-3-phenylpropanoic acid* (**5**). Yellow amorphous powder, quantitative yield. ^1^H NMR (600 MHz, DMSO-d^6^) δ 12.89 (s, 1H), 11.25 (s, 1H), 8.17 (d, *J* = 8.4 Hz, 1H), 8.10 (d, *J* = 7.2 Hz, 1H), 7.67–7.66 (m, 3H), 7.36–7.29 (m, 4H), 7.27–7.22 (m, 1H), 7.01 (s, 1H), 6.64 (s, 1H), 4.23–4.16 (m, 1H), 3.09 (dd, *J* = 5.4 and 13.5 Hz, 1H), 2.96 (dd, *J* = 9.6 and 13.8 Hz, 1H); ^13^C NMR (150 MHz, DMSO-d^6^) δ 182.2, 172.9, 163.5, 157.5, 153.7, 153.4, 153.0, 137.7, 132.1, 130.7, 129.2, 129.2, 128.2, 126.5, 126.5, 122.8, 104.8, 104.0, 94.0, 55.8, 36.6. HRMS (ESI) *m/z* calculated for C_25_H_19_NO_8_^+^ [M+H]^+^: 462.1184; found: 462.1190.

*(S)-2-((((5,6-Dihydroxy-4-oxo-2-phenyl-4H-chromen-7-yl)oxy)carbonyl)amino)propanoic acid* (**6**). Yellow amorphous powder, quantitative yield. ^1^H NMR (600 MHz, DMSO-d^6^) δ 12.93 (s, 1H), 11.27 (s, 1H), 8.11 (d, *J* = 7.8 Hz, 1H), 8.08 (d, *J* = 7.2 Hz, 2H), 7.62 (t, *J* = 7.2 Hz, 1H), 7.58 (t, *J* = 7.2 Hz, 1H), 7.00 (s, 1H), 6.66 (s, 1H), 4.09–4.02 (m, 1H), 1.33 (d, *J* = 7.2 Hz, 3H); ^13^C NMR (150 MHz, DMSO-d^6^) δ 182.2, 174.0, 163.5, 157.5, 153.7, 153.2, 153.0, 132.1, 130.7, 129.2, 126.5, 122.9, 104.9, 104.0, 94.1, 49.5, 17.3. HRMS (ESI) *m/z* calculated for _19_H_15_NO_8_^+^ [M+H]^+^: 386.0871; found: 386.0877.

*2-((((5,6-Dihydroxy-4-oxo-2-phenyl-4H-chromen-7-yl)oxy)carbonyl)amino)acetic acid* (**7**). Yellow amorphous powder, quantitative yield. ^1^H NMR (600 MHz, DMSO-d^6^) δ 12.94 (s, 1H), 11.29 (s, 1H), 8.09 (d, *J* = 7.2 Hz, 2H), 8.05 (t, *J* = 6.0 Hz, 1H), 7.62 (t, *J* = 7.8 Hz, 1H), 7.58 (t, *J* = 7.8 Hz, 2H), 7.01 (s, 1H), 6.66 (s, 1H), 3.76 (d, *J* = 6.0 Hz, 2H); ^13^C NMR (150 MHz, DMSO-d^6^) δ 182.2, 171.2, 163.5, 157.5, 153.9, 153.7, 153.0, 132.1, 130.7, 129.2, 126.5, 122.9, 104.9, 104.0, 94.1, 42.4. HRMS (ESI) *m/z* calculated for C_18_H_13_NO_8_^+^ [M+H]^+^: 372.0714; found: 372.0722.

### 2.5. Chemical Stability

Stock solutions of the test compounds (baicalein, compounds **1–7**) prepared in MeOH at 10 mM were diluted in 0.1 N HCl and 0.1 M potassium phosphate buffer (pH 6.5 and 7.4) in triplicate to a final concentration of 10 μM. The mixtures were then incubated at 37 °C in a shaking water bath. Samples taken at 0, 1, 2, 3 and 4 h were analyzed by Thermo UltiMate 3000 series HPLC system. Chromatographic separation was performed on an Agilent Eclipse Plus C18 column (100 × 4.6 mm, 3.5 µm) kept at 40 °C with isocratic elution at a flow rate of 0.6 mL/min using a mixture of 0.1 vol% TFA in deionized water and acetonitrile as the mobile phase. The detection wavelengths were 277 and 270 nm for baicalein and compounds **1–7**, respectively. Peak areas of the analytes on the UV chromatograms were used to calculate % remaining values at each time points.

### 2.6. Solubility

Stock solutions of the test compounds (baicalein and compounds **1–7**) prepared at 10 mM in DMSO were diluted with 0.1 M potassium phosphate buffer (pH 7.4) in Mini-UniPrep filter vial (PTFE membrane, 0.45 μm) to a final concentration of 500 µM. The mixture was then shaken at 800 rpm for 90 min at rt and filtered by slowly pressing the upper tube of the Mini-Uniprep vial. The filtrate (300 µL) was then transferred to an HPLC vial containing same volume of acetonitrile. Sample analysis was conducted as described above in Chemical stability assay.

### 2.7. logD_7.4_

The assay was conducted as reported previously [14]. Briefly, 100 µM test solutions were prepared by diluting 10 mM stock solutions of the test compounds (baicalein and compounds **1–7**) in 0.1 M potassium phosphate buffer (pH 7.4) saturated with 1-octanol. Standard solutions were prepared by diluting each test solution with the same volume of DMSO in HPLC vials. Partition solutions were prepared by mixing each test solution and 1-octanol saturated with 0.1 M potassium phosphate buffer (pH 7.4) at three different volume ratios, namely 1:0.02, 1:0.2 and 1:2, in HPLC vials. The partition solutions were then vortexed for 1 h at 1500 rpm at rt. The standard solutions and the aqueous phase (bottom layer) of each partition solution were analyzed directly from the HPLC vials as described above in Chemical stability assay. The *logD_7.4_* values were calculated by the following equation:

$$logD_{7.4}=log\left((2\times \frac{A_{s}}{A_{p}}-1)\cdot \frac{V_{t}}{V_{o}}\right)$$

where *A_s_* and *A_p_* are, respectively, the peak areas of the standard and the aqueous phase of the partition solutions and *V_t_* and *V*_o_ the volumes of test solution and 1-octanol of the partition solutions.

### 2.8. PAMPA

The assay was conducted as reported previously [15]. The samples were analyzed as described below in mouse plasma stability assay. Low permeability references (atenolol, hydrochlorothiazide, nadolol and ranitidine) had *P_app_* values <1 nm/sec, while high permeability references (chloramphenicol, ketoprofen, metoprolol, propranolol and verapamil) showed *P_app_* values >10 nm/sec.

### 2.9. Metabolic Stability

#### 2.9.1. Mouse Plasma

Working solutions of the test compounds (baicalein, enalapril, and compounds **1–7**) prepared in 50 vol% aqueous MeOH at 40 μM were diluted in mouse plasma in triplicate to a final concentration of 1 μM. The mixtures were then incubated at 37 °C in a shaking water bath. A 50 μL aliquot was removed at 0 and 60 min from each incubation mixture followed by immediate mixing with 150 μL ice-cold MeOH containing 1 μg/mL glipizide as the internal standard (IS). The resulting mixtures, following a 5-min vortexing and sonication, were then centrifuged at 3000 rpm for 30 min at 4 °C. The supernatant was analyzed by Agilent 6460 QQQ LC−MS/MS system in a positive multiple reaction monitoring (MRM) mode. Gradient elution was conducted on an Agilent Eclipse Plus C18 column (100 × 2.1 mm, 3.5 µm) at 40 °C with 0.1 vol% formic acid in deionized water and 0.1 vol% formic acid in acetonitrile running at 0.45 mL/min. MRM transitions of the analytes were set as follows: baicalein: *m/z* 271→123, enalapril: *m/z* 377→234, 1: *m/z* 428→271, 2: *m/z* 414→271, 3: *m/z* 428→271, 4: *m/z* 416→271, 5: *m/z* 462→271, 6: *m/z* 386→271, 7: *m/z* 372→271, and glipizide: *m/z* 446→321. The analytical data were analyzed using Agilent MassHunter Quantitative Analysis Software (version B.05.00). Peak area ratios of the analytes vs. IS on the MRM chromatograms were used to calculate % remaining at 60 min.

#### 2.9.2. Mouse Liver S9 Fractions

Working solutions of the test compounds (baicalein, 7-EC, and compounds **1–7**) prepared in 50 vol% aqueous MeOH at 120 μM were added in triplicate to a 200 mM tris buffer (pH 7.4) containing mouse liver S9 fractions (1 mg protein/mL), alamethicin (25 μg/mL) and MgCl_2_ (2 mM). The final concentration of test compound was 3 μM. This mixture was pre-incubated at 37 °C in a shaking water bath for 5 min followed by addition of a cofactor mixture to following final concentrations: 1 mM NADPH, 0.5 mM UDPGA, 0.1 mM PAPS and 2.5 mM GSH. A 50 μL aliquot was taken at 0, 15, 30 and 60 min from each incubation mixture. These samples were then treated in the same way as described above in Mouse plasma stability assay. The supernatant was analyzed by Agilent 6530 Q−TOF LC−MS/MS system in a positive auto MS/MS scan mode. The liquid chromatography was conducted in the same condition as described above in mouse plasma stability assay. The analytical data were analyzed using Agilent MassHunter Quantitative Analysis Software (version B.05.00). MS chromatograms were extracted using calculated exact masses ([M+H]^+^) for the analytes. Peak area ratios of the analytes vs. IS on the extracted MS chromatograms were used to calculate % remaining values at each time points.

#### 2.9.3. Mouse Pharmacokinetics

Pharmacokinetic studies were conducted with male ICR mice (8 weeks old; 30–35 g body wt.) purchased from Koatech Company (Pyeongtaek, Korea), as reported previously [16]. Intravenous dosing solutions were prepared in EtOH/20% aqueous HPβCD (10/90 vol%) at 2 and 3 mg/mL for baicalein and compound **2**, respectively. Oral dosing solutions were prepared in EtOH/tween 80/20% aqueous HPβCD (10/10/80 vol%) at 1 and 1.5 mg/mL for baicalein and compound **2**, respectively. Plasma sample analysis was performed as described above in mouse plasma stability assay.

## 3. Results and Discussion

### 3.1. Synthesis

**Scheme 1:**
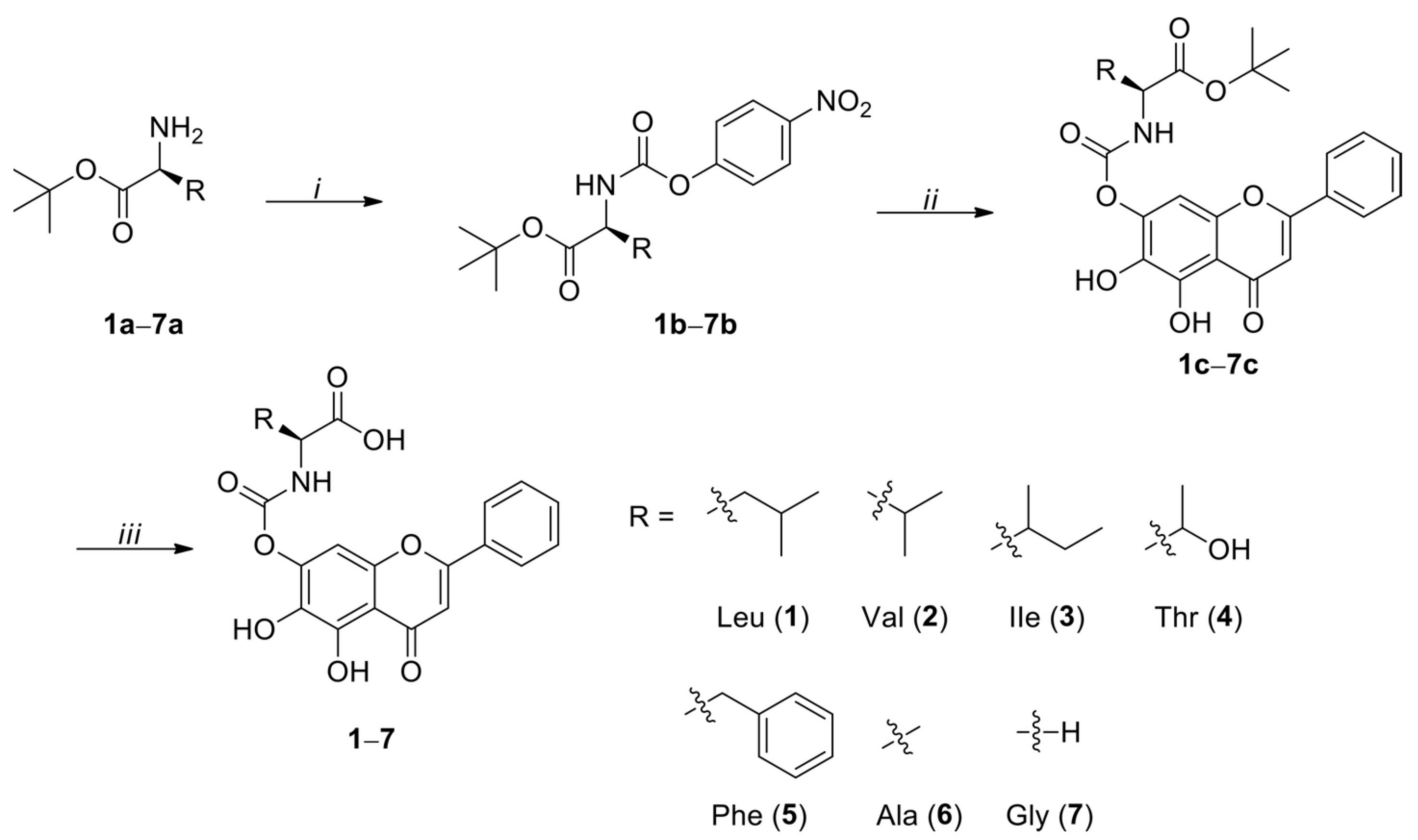
Synthesis of baicalein carbamate prodrugs. Reagent and conditions: (*i*) bis-(4-nitrophenyl)carbonate, DIPEA, THF, rt, 12 h; (*ii*) baicalein, DIPEA, THF, rt, 12 h; (*iii*) 25% TFA in CH_2_Cl_2_, rt, 6 h.

The carbamate functional group is generally more stable than the corresponding ester group because the carbonyl carbon atom is less electrophilic in carbamates than esters. Therefore, we aimed to introduce the carbamate functional group at the 7-position of baicalein. Without the protection of the OH groups at the 5- and 6-position, we could selectively conjugate the amino acid with the -OH group at the 7-position of baicalein. In the 2D NOESY NMR experiment with compound **1c**, the cross-peak between H-8 and N-H of the carbamate group was observed (See Supplementary Materials). The synthetic route for baicalein carbamate prodrugs is shown in Scheme 1. Briefly, various amino acids (**1a–7a**) in which the carboxylic acid is protected with a *tert*-butyl group, were reacted with bis-(4-nitrophenyl)carbonate under basic conditions to provide the activated carbamate analogs (**1b–7b**). Under the basic condition (diisopropylethylamine (DIPEA) in THF), the 7-OH group of baicalein reacted with the activated carbamate compounds to form the baicalein carbamate analogs **1c–7c** in high yields (47–80%). Deprotection of the *tert*-butyl group was performed in 25% trifluoroacetic acid in dichloromethane to give the final compounds **1–7** in quantitative yield.

### 3.2. Chemical Stability

The stability of baicalein and of the synthesized carbamate prodrugs (**1–7**) was determined in solutions mimicking gastric (0.1 N HCl, pH ~ 1), intestinal (pH 6.5 phosphate buffer), and physiological (pH 7.4 phosphate buffer) conditions. All test compounds were relatively stable, with a *t_1/2_* greater than 1 h in the solutions used in this study (Table 1). Baicalein showed a slow degradation, with 65.8% remaining after 4 h incubation at pH 7.4, but it remained intact at a lower pH (i.e., 0.1 N HCl and pH 6.5 buffer) (Table 1). Some carbamate prodrugs also showed pH-dependent stability, distinct from baicalein. Compounds **1**, **5**, and **7** were relatively unstable in 0.1 N HCl with a *t_1/2_* of 1.4–2.2 h; all the carbamate prodrugs were stable at higher pH, with a t_1/2_ greater than 4 h, except for compound **4** (Table 1). Compound **4** was less stable at pH 6.5 and 7.4 compared with 0.1 N HCl (Table 1); the OH group of the Thr side chain may be responsible for this unique stability profile, as compound **4** was the only test compound with an OH group in the pro-moiety. The % remaining values greater than 100 were likely due to variabilities in the experimental procedure and the analytical method. The degradation of the carbamate prodrugs was accompanied by the formation of baicalein under all conditions in this study (data not shown). Although the exact reason for this pH-dependent stability remains unknown, it is plausible that the side chains of amino acids somehow affect the hydrolysis of the carbamate bond of the prodrugs. These results also suggest that carbamate prodrugs of baicalein may be suitable as oral therapeutic agents depending on the amino acid incorporated as a pro-moiety.

### 3.3. Physicochemical Properties

Various physicochemical properties of baicalein and carbamate prodrugs were either calculated or measured in this study to assess their feasibility as drug-like compounds.

The conjugation of the amino acids to baicalein naturally increased their molecular weight (*MW*) by ~101–191 (Table 2); however, their *MW*s were still less than 500, conforming to the Lipinski’s rule-of-five. The topological polar surface area (*TPSA*) also increased significantly due to the polar atoms present in the pro-moiety exceeding even the widely accepted cutoff of 140 Å^2^ (Table 2), suggesting that the prodrugs may have a significantly lower membrane permeability than baicalein. In fact, the apparent permeability coefficient (*P_app_*) measured by the parallel artificial membrane permeability assay (PAMPA) for compounds **2** and **6** was low, in contrast to the high permeability of baicalein (Table 2). The calculated *logP* values of baicalein and the prodrugs were all within a comparable range that was lower than the rule-of-five cutoff of five (Table 2).

On the contrary, *logD_7.4_* values of the prodrugs (determined in this study by the shake-flask method) were significantly lower than that of baicalein (Table 2), likely due to the ionized carboxylate group of the amino acids and consistent with the difference in the membrane permeability described above. The kinetic solubility of the prodrugs determined in phosphate buffer (pH 7.4), similar to *logD_7.4_*, was much higher than that of baicalein, consistent with the observed decrease in lipophilicity (*logD_7.4_*) (Table 2). Based on these results, the introduction of the amino acid pro-moiety to baicalein appears to increase the aqueous solubility at the cost of membrane permeability.

### 3.4. Bioconversion and Metabolic Stability

Biotransformation of carbamate prodrugs to baicalein was investigated in the plasma and liver S9 fractions representing the tissues responsible for prodrug bioconversion in general in the body. Mice were selected for both in vitro and in vivo evaluations in this study, as this is the most frequently used animal model for asthma [17] and atopic dermatitis [18].

**Figure 2:**
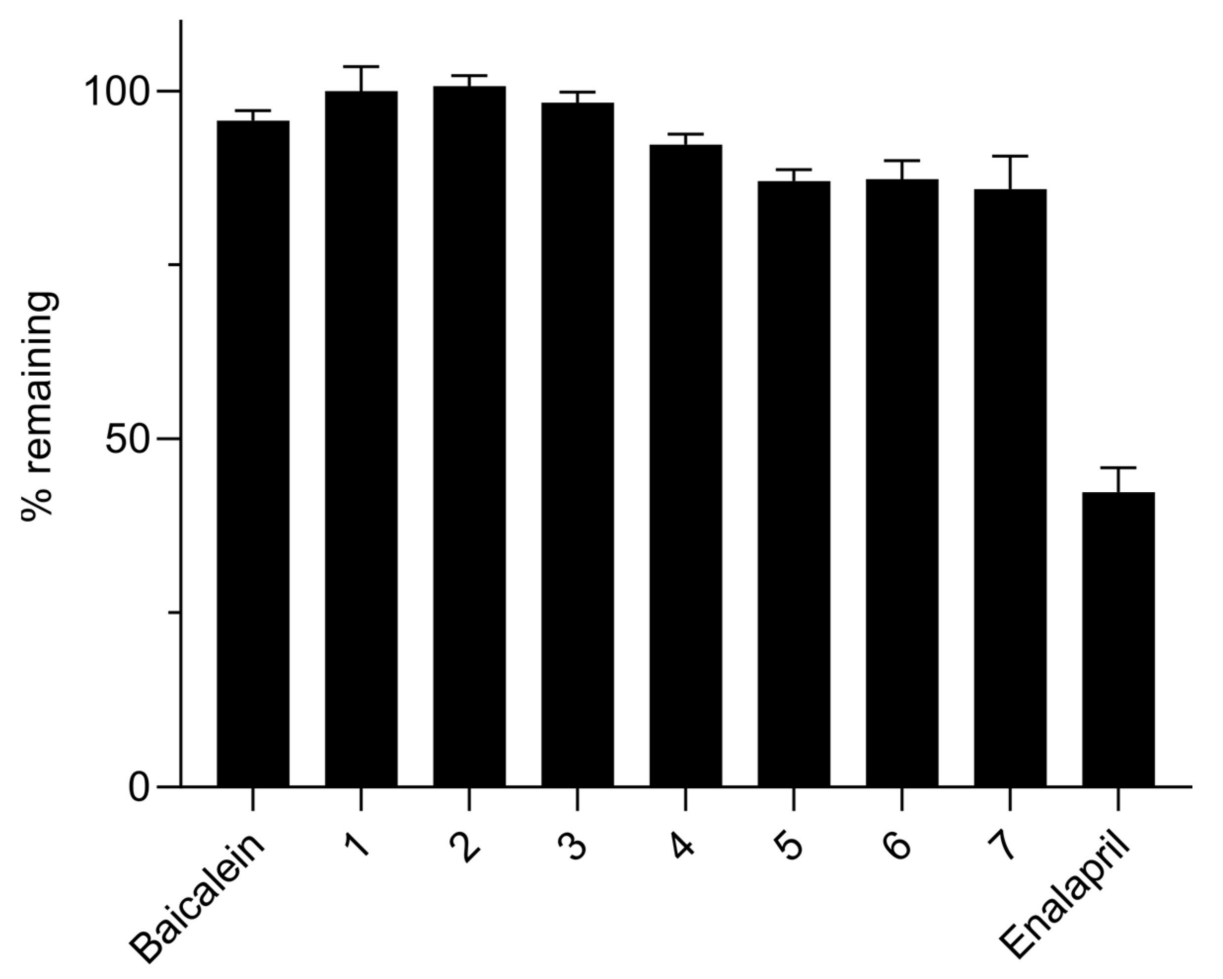
Stability of baicalein and its carbamate prodrugs in mouse plasma. Test compounds were incubated for 60 min at 37 °C in mouse plasma. % remaining was determined at 60 min vs. time 0 using peak ratios of LC−MS/MS chromatograms. Data are expressed as the mean ± SD (*n* = 3).

Baicalein and the prodrugs were generally stable in mouse plasma, with >85% remaining after 60 min incubation at 37 °C (Figure 2); baicalein was not detected in any of the prodrug incubation mixtures (data not shown). Enalapril, included as a control for hydrolase activity [19], was degraded as expected (Figure 2). These results suggest that plasma may not be the main tissue responsible for the bioconversion of carbamate prodrugs in the body.

**Figure 3:**
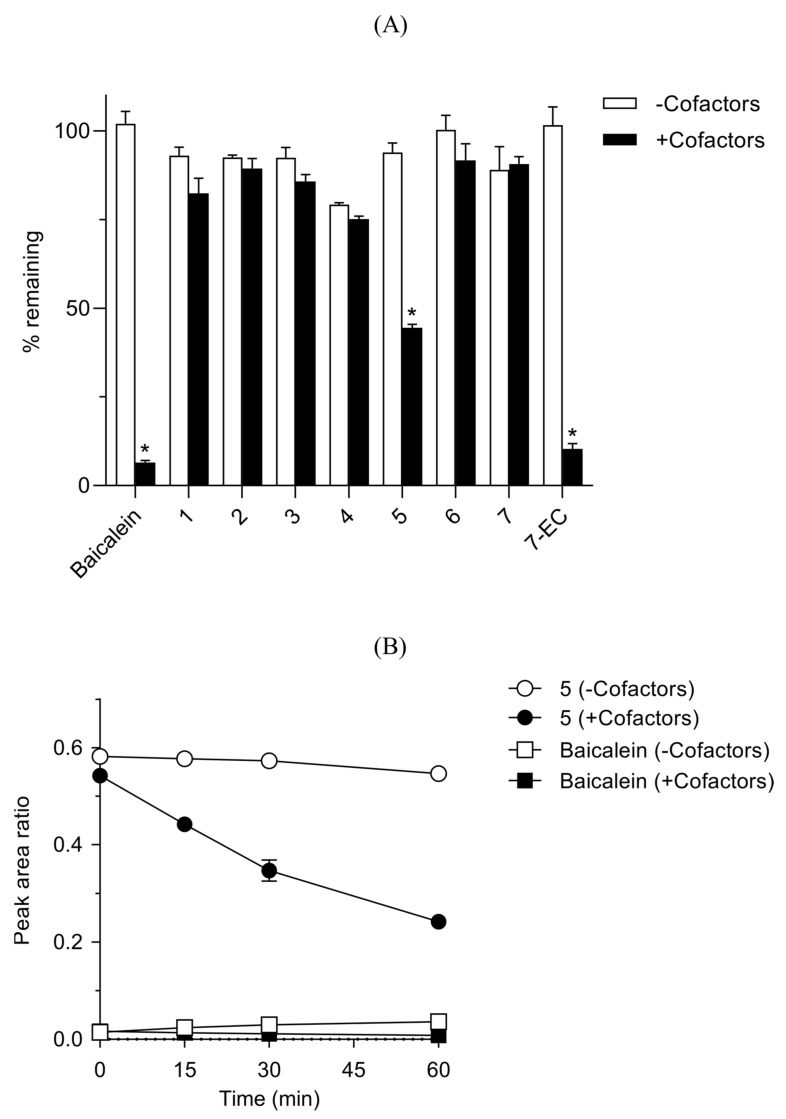
Metabolic stability of baicalein and its carbamate prodrugs in the mouse liver S9 fractions. Test compounds were incubated at 37 °C with mouse liver S9 fractions in the absence and presence of the cofactor mixture (NADPH, UDPGA, PAPS and GSH). (**A**) % remaining was determined at 60 min vs. time 0 using peak ratios of LC−MS/MS chromatograms (mean ± SD, *n* = 3). * *p* < 0.001 (multiple unpaired *t* test). (**B**) Time course of compound **5** disappearance and its conversion to baicalein was determined simultaneously in the mouse liver S9 fractions following incubation of compound **5** in the absence and presence of the cofactor mixture (mean ± SD, *n* = 3).

The metabolic stability and bioconversion of the test compounds in mouse liver S9 fractions were fortified with cofactors required for major drug-metabolizing enzymes, including nicotinamide adenine dinucleotide phosphate (NADPH), CYP450, uridine 5′-diphosphoglucuronic acid (UDPGA, for UDP-glucuronosyltransferase), 3-phosphoadenosine-5′-phosphosulfate (PAPS, for sulfotransferase), and glutathione (GSH, for glutathione S-transferase). Baicalein, as reported in the literature [7], was rapidly metabolized in the presence of the cofactor mixture (Figure 3A). The prodrugs tested were all relatively stable, with >75% remaining after 60 min incubation at 37 °C in both the absence and presence of the cofactors, except for compound **5** (Figure 3A). Compound **5** was metabolized much faster than the other prodrugs in the presence of the cofactors (Figure 3A); however, its metabolism was not accompanied by a corresponding increase in baicalein levels (Figure 3B). It is plausible that an aromatic oxidation in the Phe pro-moiety of compound **5** was responsible for its rapid metabolism as it was the only test compound with an aromatic side chain among compounds **1–7**. 7-Ethoxycoumarin (7-EC), included as a control for metabolic activity, showed rapid metabolism in the presence of the cofactors, as expected (Figure 3A). These results suggest that the liver may not serve as the main tissue responsible for the bioconversion of carbamate prodrugs in the body. Compound **5** may be metabolized by biotransformation reactions other than carbamate hydrolysis, or baicalein formed from carbamate hydrolysis may be further metabolized rapidly, as demonstrated with baicalein itself.

### 3.5. Pharmacokinetics

Pharmacokinetic studies were conducted in vivo with baicalein and compound **2**, a representative carbamate prodrug, in mice in order to investigate whether the carbamate prodrugs improve the pharmacokinetic behavior of baicalein, especially the plasma exposure, following intravenous (i.v.) and oral (p.o.) administration. Compound **2** was chosen as the model compound for in vivo studies because it showed the highest PAMPA permeability among the prodrugs that were stable in all the physiologically-relevant solutions tested in this study (Table 1 and Table 2).

Plasma baicalein was above the quantitation limit only up to 15 min following a single i.v. dose of 10 mg/kg baicalein in mice (Table 3); the primary pharmacokinetic parameters requiring the terminal loglinear phase, such as the steady-state volume of distribution (*V_ss_*) and systemic clearance (*CL*), could not be determined for baicalein in this study. Baicalin, on the other hand, was present above the quantitation limit in the plasma for 24 h following the i.v. dose of baicalein, and its plasma area under the curve (*AUC*) was much higher than that of the parent (Table 3). Plasma baicalein was below the quantitation limit throughout the time course for 24 h post-dosing following a single p.o. dose of 10 mg/kg baicalein in mice (Table 3), and baicalin was detected in the plasma above the quantitation limit for 24 h, with its plasma *AUC* even higher than that from the i.v. dosing (Table 3), indicating a significant first-pass metabolism during oral absorption [7]. On the contrary, compound **2**, when administered i.v. at an approximately equimolar dose of 10 mg/kg baicalein, resulted in a much higher plasma *AUC* due to its low *V_ss_* and *CL* (Table 3). More importantly, baicalein was detected in the plasma above the quantitation limit for 8 h post-dosing, and its plasma *AUC* was also much higher compared to that following the i.v. dose of the parent (Table 3). These results clearly demonstrate that the prodrug was converted to the parent drug in the body. The p.o. administration of compound **2** produced a much lower plasma exposure compared to the i.v. dosing, thereby giving a low oral bioavailability (Table 3); again, baicalein was detected in the plasma above the quantitation limit for 8 h, giving a much higher plasma *AUC* compared with the parent dosing (Table 3) and suggesting that the carbamate prodrug significantly improved the oral delivery of baicalein.

As shown in our in vitro studies, the liver and plasma are not likely to be the tissues where bioconversion of the carbamate prodrug occurs in the body. Further studies are warranted to identify the mechanism as well as the specific tissue (or organ) that acts as the site for carbamate prodrug bioconversion in the body. The poor oral bioavailability of compound 2 itself was likely due to its low intestinal permeability, as described above in the in vitro evaluations.

## 4. Conclusions

Baicalein, an active ingredient of *S. baicalensis*, has shown desirable biological activities, such as anti-inflammatory, antioxidant, and anti-tumor activities. However, the rapid clearance of baicalein through phase II metabolism in the liver limits its preclinical and clinical applications. To improve its bioavailability, we introduced the carbamate group in the OH group at the 7-position, in which the major biotransformation of baicalein takes place. Compound **2**, a baicalein prodrug conjugated to valine amino acid via the carbamate group, showed improved pharmacokinetic behavior after intravenous and oral administration when compared to baicalein itself. The amino acid carbamate approach established in this study could be used to discover and develop oral baicalein prodrugs.

## Figures and Tables

**Table 1:** Chemical stability of baicalein and its carbamate prodrugs in physiologically-relevant solutions.

Compound	0.1 N HCl		pH 6.5 Buffer		pH 7.4 Buffer	
	*t_1/2_* (h)	% Remaining	*t_1/2_* (h)	% Remaining	*t_1/2_* (h)	% Remaining
Baicalein	>4	103.6 ± 4.1	>4	101.3 ± 2.8	>4	65.8 ± 2.6
**1**	1.4 ± 0.3	17.4 ± 2.1	>4	92.9 ± 5.0	>4	98.0 ± 3.6
**2**	>4	105.2 ± 3.5	>4	92.8 ± 4.2	>4	74.4 ± 6.6
**3**	>4	98.9 ± 5.0	>4	90.2 ± 1.1	>4	80.1 ± 2.5
**4**	>4	109.2 ± 3.4	2.8 ± 0.1	37.3 ± 0.2	1.7 ± 0.1	20.0 ± 1.0
**5**	2.2 ± 0.4	27.4 ± 5.1	>4	78.5 ± 1.5	>4	70.2 ± 2.5
**6**	>4	90.2 ± 4.1	>4	81.8 ± 1.3	>4	76.6 ± 1.7
**7**	1.5 ± 0.3	23.3 ± 3.6	>4	82.2 ± 1.0	>4	76.7 ± 0.3

Test compounds were incubated for 4 h at 37 °C in 0.1 N HCl and 0.1 M potassium phosphate buffer (pH 6.5, or 7.4). % remaining was determined at 4 h versus time 0 using peak ratios of liquid chromatography−tandem mass spectrometry (LC−MS/MS) chromatograms. Data are expressed as the mean ± standard deviation (SD) (*n* = 3).

**Table 2:** Physicochemical properties of baicalein and its carbamate prodrugs.

Compound	*MW*	*TPSA*^a^ (Å^2^)	*logP* ^b^	*logD_7.4_* ^c^	Solubility ^d^ (μM)	*P_app_*^e^ (nm/sec)
Baicalein	270.2	87.0	1.90	2.14	62.0 ± 9.1	57.5 ± 1.4
**1**	427.4	142.4	2.78	0.39	442.7 ± 65.9	ND
**2**	413.4	142.4	2.43	0.04	479.2 ± 4.4	0.4 ± 0.2
**3**	427.4	142.4	2.85	0.25	447.3 ± 36.9	ND
**4**	415.4	162.6	1.01	-0.05	346.8 ± 11.4	ND
**5**	461.4	142.4	3.22	0.43	375.0 ± 21.4	ND
**6**	385.3	142.4	1.55	-0.18	400.3 ± 14.7	<0.1
**7**	371.3	142.4	1.05	-0.39	357.1 ± 1.4	ND

^a^ Calculated by ChemBioDraw Ultra 14.0; ^b^ Calculated by ChemBioDraw Ultra 14.0; ^c^ Determined in duplicate by the shake-flask method in 0.1 M phosphate buffer (pH 7.4); ^d^ Kinetic solubility was determined in triplicate by the shake-flask method (mean ± SD) in 0.1 M phosphate buffer (pH 7.4); ^e^ Determined in triplicate by PAMPA (mean ± SD) as described in the literature [15]. ND: Not determined.

**Table 3:** Pharmacokinetics of baicalein and compound **2** in mice.

Parameter	Baicalein		Compound 2		
	Baicalein	Baicalin	Compound 2	Baicalein	Baicalin
i.v.					
*Dose* (mg/kg)	10	NA	15	NA	NA
*t_max_* (h)	0.1 ± 0.0	0.1 ± 0.0	0.1 ± 0.0	0.1 ± 0.0	0.1 ± 0.0
*C_max_* (μg/mL)	1.47 ± 0.33	4.59 ± 0.87	65.07 ± 4.29	4.19 ± 0.45	0.09 ± 0.01
*AUC_last_* (μg⋅h/mL)	1.09 ± 0.64	2.45 ± 0.49	88.6 ± 43.2	4.24 ± 1.43	0.20 ± 0.088
*AUC_inf_* (μg h/mL)	ND	2.60 ± 0.51	88.70 ± 43.18	4.39 ± 1.57	0.50 ± 0.17
*V_SS_* (l/kg)	ND	NA	0.4 ± 0.1	NA	NA
*CL* (l/h/kg)	ND	NA	0.2 ± 0.1	NA	NA
*t_1/2_* (h)	ND	6.2 ± 2.8	1.9 ± 0.6	1.5 ± 0.3	6.6 ± 1.6
p.o.					
*Dose* (mg/kg)	10	NA	50	NA	NA
*t_max_* (h)	ND	0.2 ± 0.0	0.3 ± 0.09	0.3 ± 0.09	6.0 ± 2.8
*C_max_* (μg/mL)	ND	2.26 ± 0.67	2.93 ± 0.59	0.23 ± 0.09	0.13 ± 0.04
*AUC_last_* (μg h/mL)	ND	4.42 ± 1.44	5.43 ± 1.73	0.45 ± 0.14	0.68 ± 0.21
*AUC_inf_* (μg⋅h/mL)	ND	4.89 ± 1.40	5.82 ± 1.71	0.54 ± 0.15	ND
*t_1/2_* (h)	ND	6.3 ± 3.0	1.7 ± 0.5	2.6 ± 1.5	ND
*F* (%)	ND	NA	2.0 ± 0.6	NA	NA

Pharmacokinetic parameters were calculated using PKSolver [20] by non-compartmental analysis of the plasma concentration-time curves obtained following a single dose of baicalein or compound **2** in mice. Values are presented as the mean ± SD (*n* = 4). NA: not applicable. ND: not determined.

## Data Availability

Data is contained within the article.

## Supplementary Materials

The following are available online at https://www.mdpi.com/article/10.3390/pharmaceutics13091516/s1, Figure S1. ^1^H NMR spectra of compound **1c**. Figure S2. ^13^C NMR spectra of compound **1c**. Figure S3. ^1^H NMR spectra of compound **1c** measured in DMSO before the treatment of D_2_O at 600 MHz. Figure S4. ^1^H NMR spectra of compound **1c** measured in DMSO after the treatment of D_2_O at 600 MHz. Figure S5. ^1^H NOESY NMR spectra of compound **1c** measured in DMSO at 600 MHz. Figure S6. ^1^H NMR spectra of compound **2c**. Figure S7. ^13^C NMR spectra of compound **2c**. Figure S8. ^1^H NMR spectra of compound **3c**. Figure S9. ^13^C NMR spectra of compound **3c.** Figure S10. ^1^H NMR spectra of compound **4c**. Figure S11. ^13^C NMR spectra of compound **4c**. Figure S12. ^1^H NMR spectra of compound **5c**. Figure S13. ^13^C NMR spectra of compound **5c**. Figure S14. ^1^H NMR spectra of compound **6c**. Figure S15. ^13^C NMR spectra of compound **6c**. Figure S16. ^1^H NMR spectra of compound **7c**. Figure S17. ^13^C NMR spectra of compound **7c**. Figure S18. ^1^H NMR spectra of compound **1**. Figure S19. ^13^C NMR spectra of compound **1**. Figure S20. HPLC chromatogram of compound **1** eluted using Method C. Figure S21. ^1^H NMR spectra of compound **2**. Figure S22. ^13^C NMR spectra of compound **2**. Figure S23. HPLC chromatogram of compound **2** eluted using Method C. Figure S24. ^1^H NMR spectra of compound **3.** Figure S25. ^13^C NMR spectra of compound **3**. Figure S26. HPLC chromatogram of compound **3** eluted using Method C. Figure S27. ^1^H NMR spectra of compound **4**. Figure S28. ^13^C NMR spectra of compound **4.** Figure S29. HPLC chromatogram of compound **4** eluted using Method B. Figure S30. ^1^H NMR spectra of compound **5.** Figure S31. ^13^C NMR spectra of compound **5**. Figure S32. HPLC chromatogram of compound **5** eluted using Method A. Figure S33. ^1^H NMR spectra of compound **6**. Figure S34. ^13^C NMR spectra of compound **6**. Figure S35. HPLC chromatogram of compound **6** eluted using Method A. Figure S36. ^1^H NMR spectra of compound **7**. Figure S37. ^13^C NMR spectra of compound **7**. Figure S38. HPLC chromatogram of compound **7** eluted using Method A.
