# Supplementary material for: Synthesis and Biochemical Evaluation of Baicalein Prodrugs
Source: Pharmaceutics. 2021 Sep 19;13(9):1516. doi: 10.3390/pharmaceutics13091516 (PMC8472512; doi:10.3390/pharmaceutics13091516)
Supplement: Supplementary file 1 [file pharmaceutics-13-01516-s001.zip › pharmaceutics-1380139-supplementary.pdf]

pharmaceutics

Supplementary Materials: Synthesis and Biochemical Evaluation of Baicalein Prodrugs

Sang-Hyun Son, Jinhong Kang, Myunghwan Ahn, Soyeon Nam, Yong Woo Jung, Ki Yong Lee, Young Ho Jeon, Youngjoo Byun and Kiho Lee

General Method of Purity Determination Using RP-HPLC

The purity of all final compounds was measured by analytical reverse-phase HPLC on an Agilent 1260 Infinity (Agilent) with a C18 column (Phenomenex, 150 mm × 4.6 mm, 3 μm, 110 Å). RP-HPLC was performed using the following isocratic conditions: for method A, the mobile phase was acetonitrile and water with 0.1% trifluoroacetic acid (TFA) (30:70, *v*/*v*); for method B, the mobile phase was acetonitrile and water with 0.1% trifluoroacetic acid (TFA) (35:65, *v*/*v*); for method C, the mobile phase was acetonitrile and water with 0.1% trifluoroacetic acid (TFA) (40:60, *v*/*v*). All compounds were eluted with a flow rate of 1 mL/min and monitored at UV detector (220 nm or 254 nm). The purity of the tested compounds was >95%.

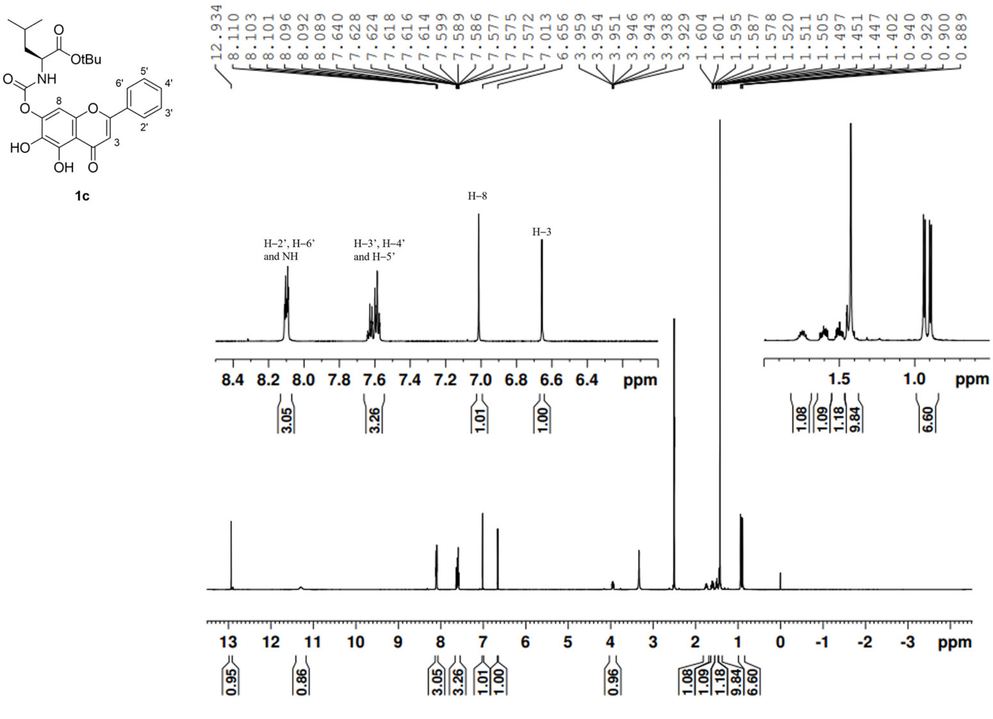

Figure S1. ^{1}H NMR spectra of compound **1c**.

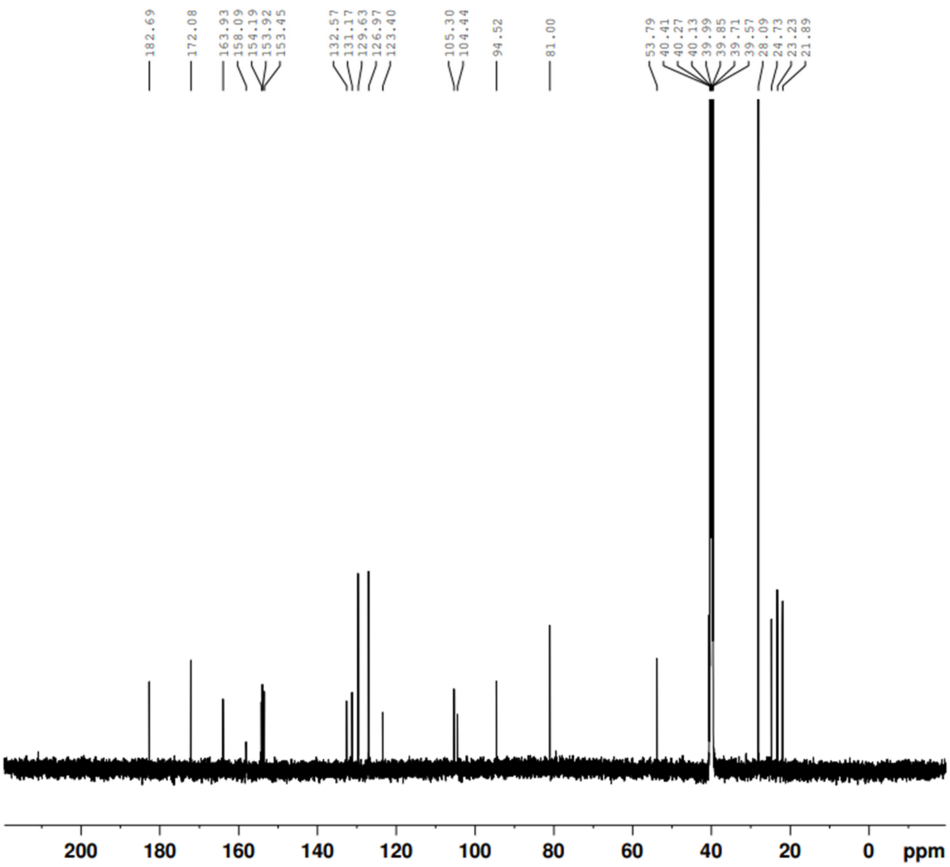

Figure S2. ^{13}C NMR spectra of compound **1c**.

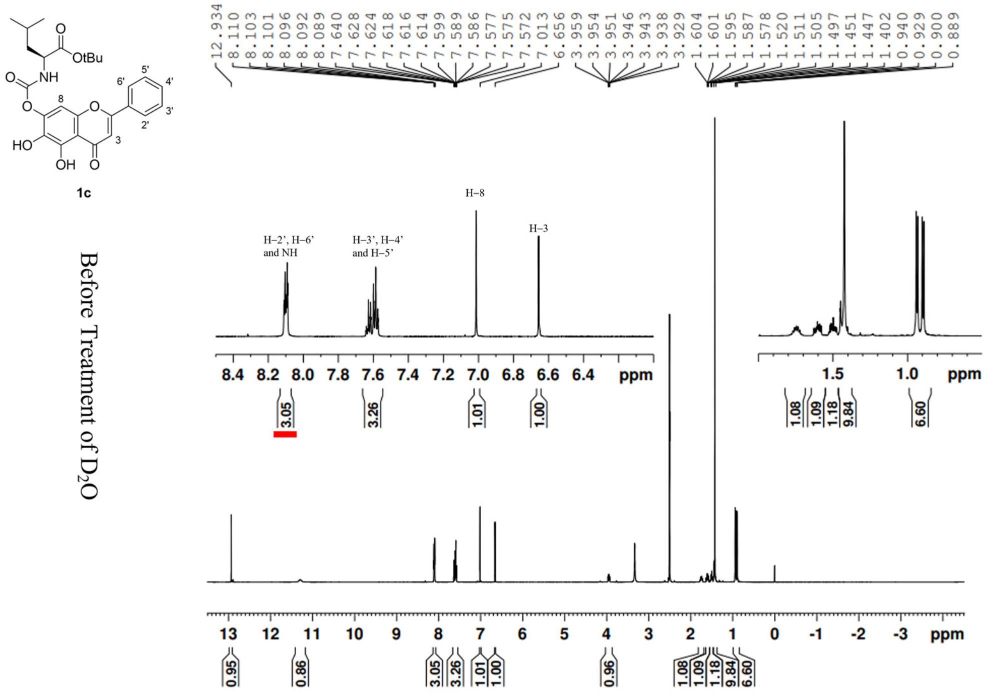

Figure S3. ^{1}H NMR spectra of compound **1c** measured in DMSO before the treatment of D_2O at 600 MHz.

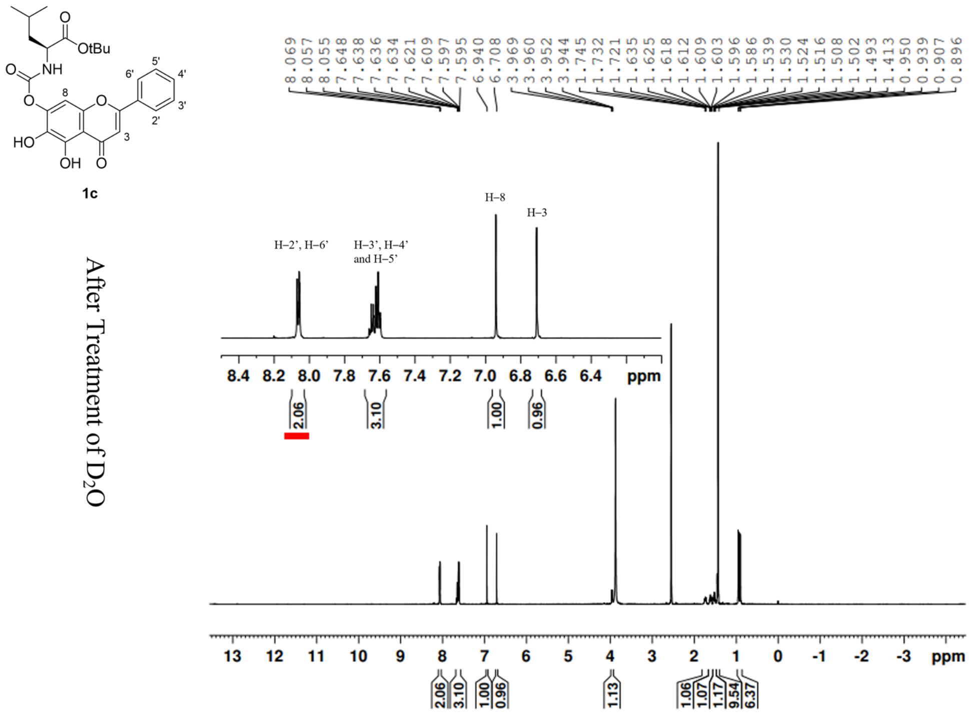

Figure S4. ^{1}H NMR spectra of compound **1c** measured in DMSO after the treatment of D_2O at 600 MHz.

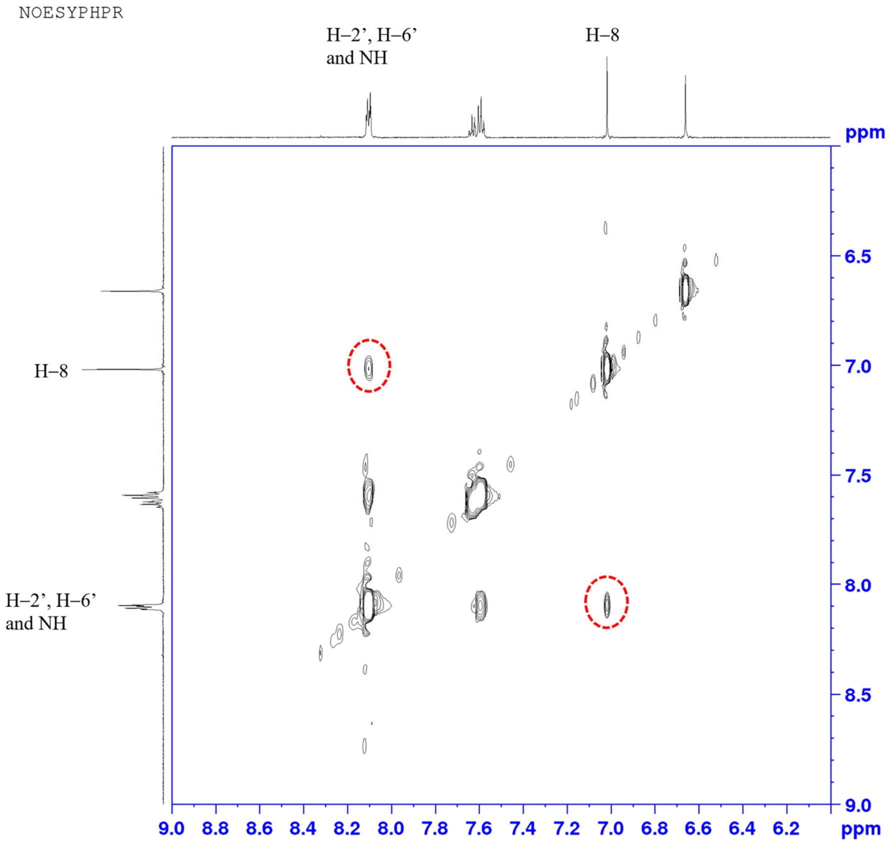

Figure S5. ¹H NOESY NMR spectra of compound **1c** measured in DMSO at 600 MHz.

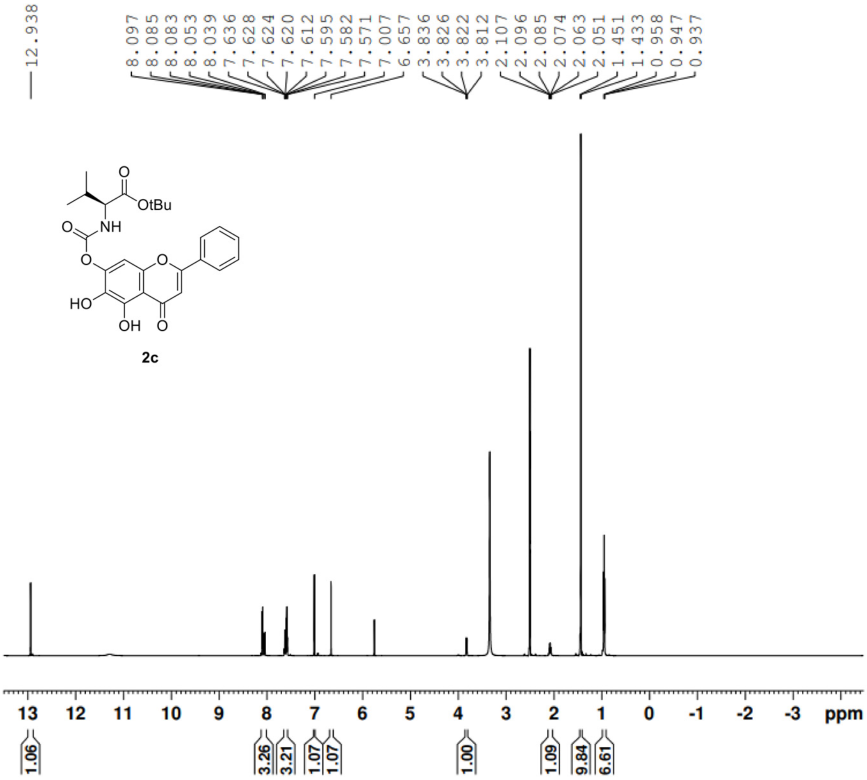

Figure S6. ¹H NMR spectra of compound **2c**.

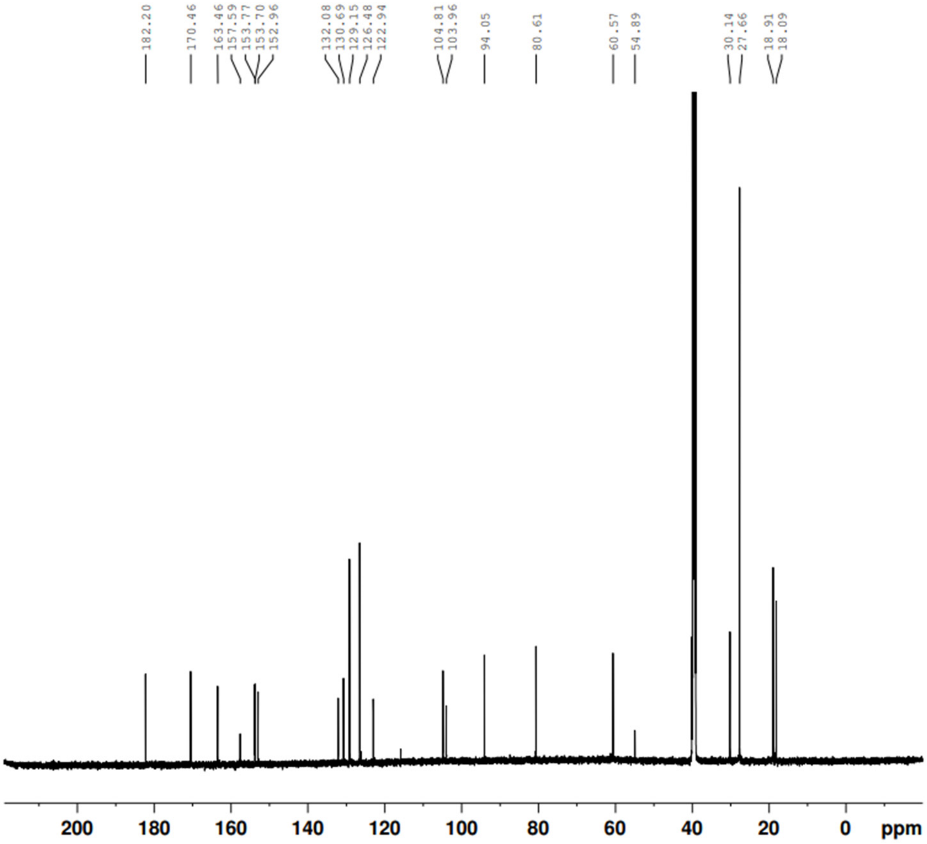

Figure S7. ^{13}C NMR spectra of compound **2c**.

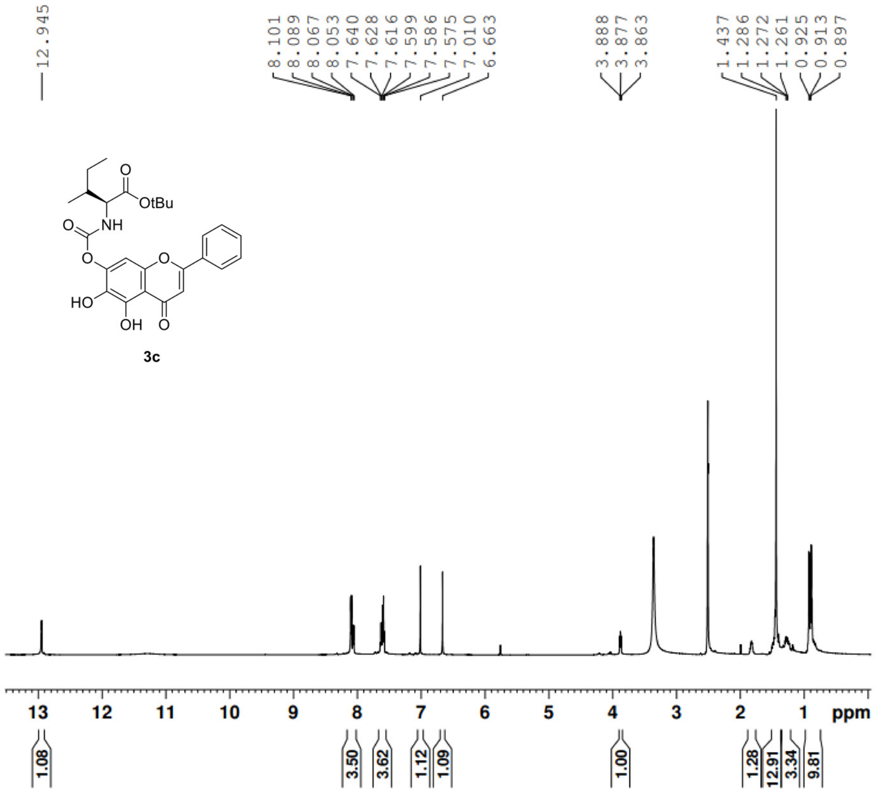

Figure S8. ^{1}H NMR spectra of compound **3c**.

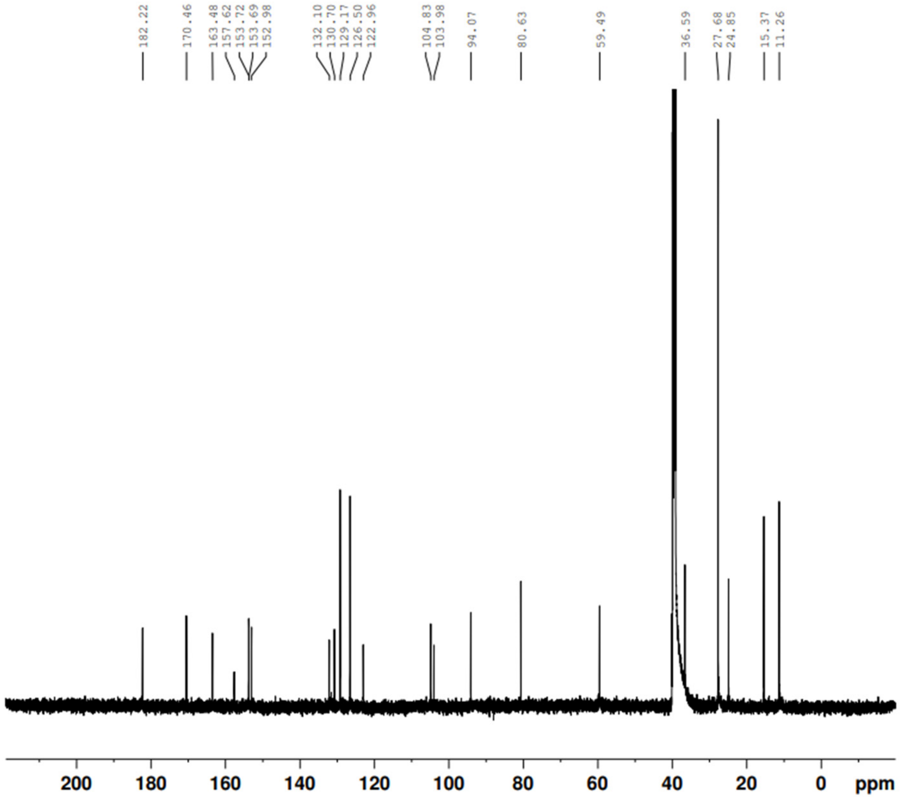

Figure S9. ^{13}C NMR spectra of compound **3c.**

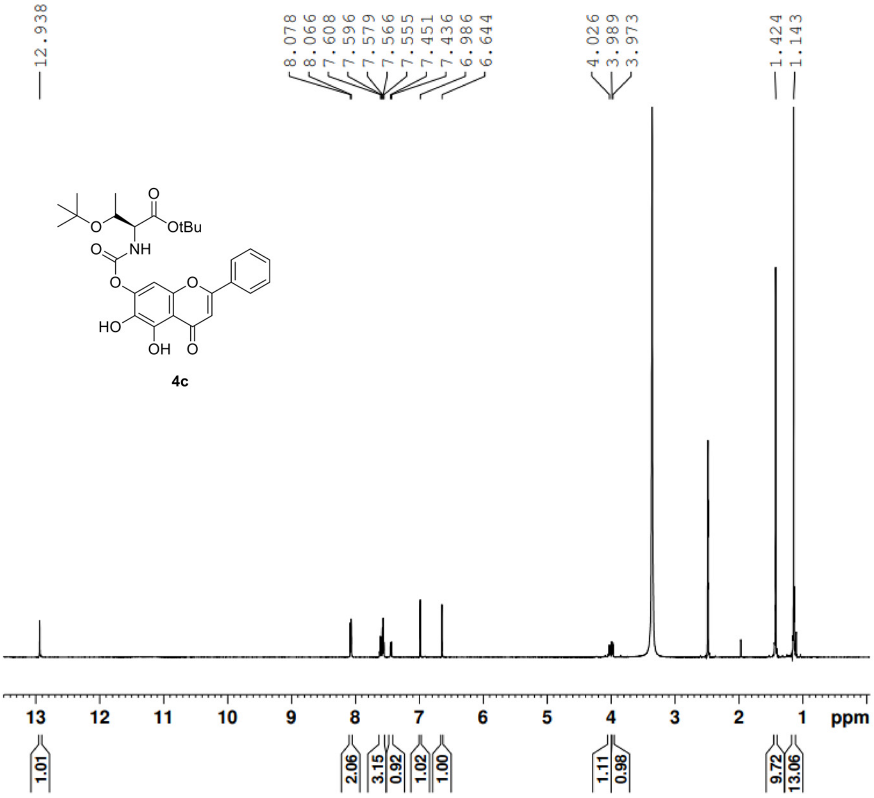

Figure S10. ^{1}H NMR spectra of compound **4c**.

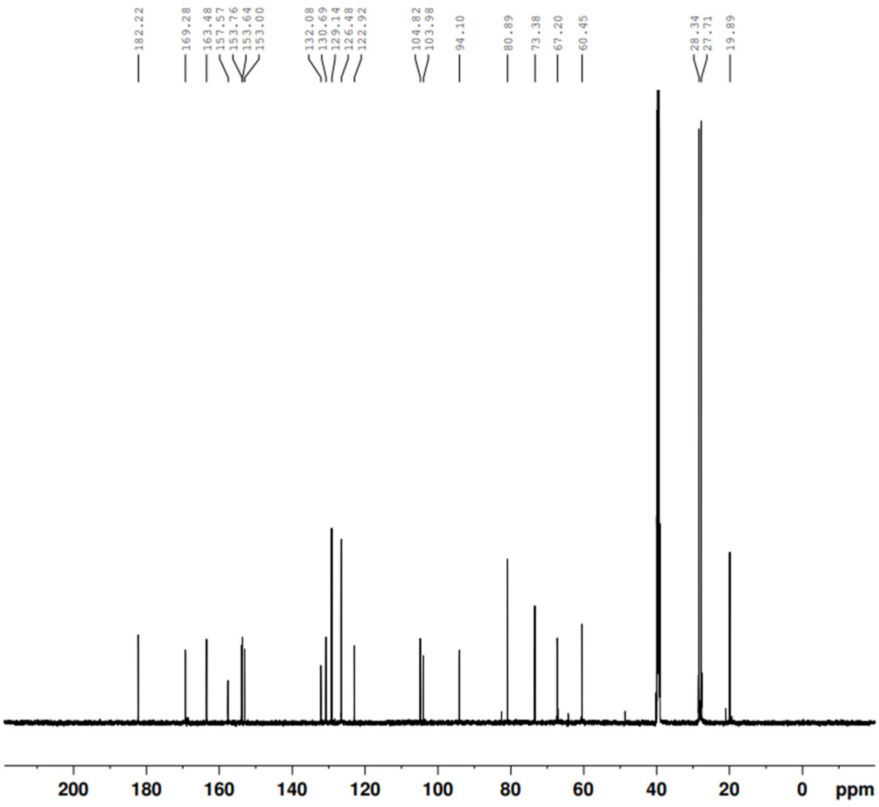

Figure S11. ^{13}C NMR spectra of compound **4c**.

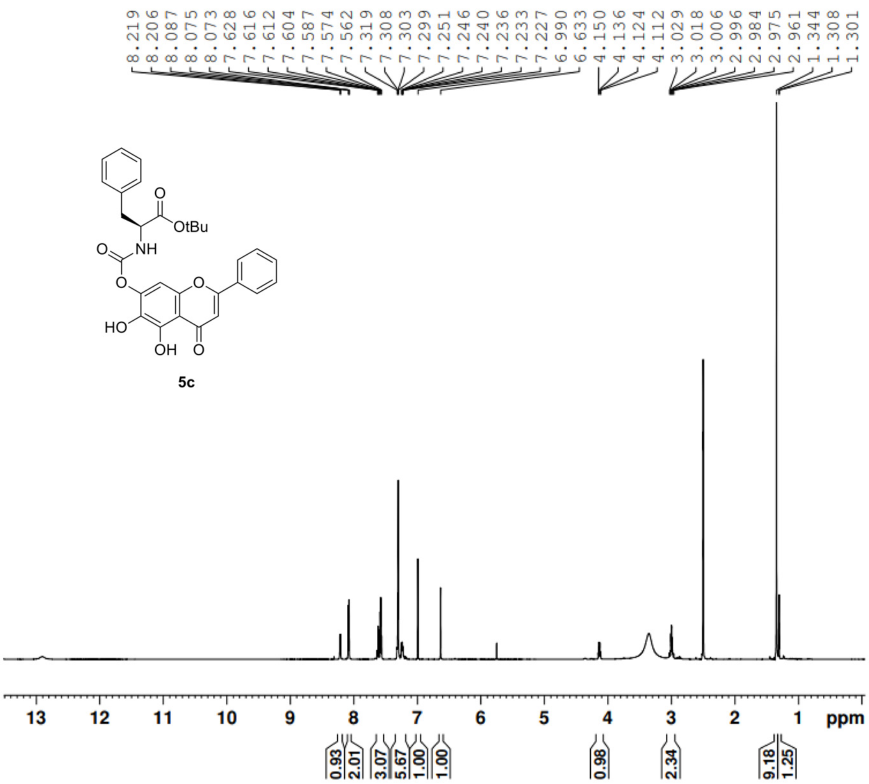

Figure S12. ^{1}H NMR spectra of compound **5c**.

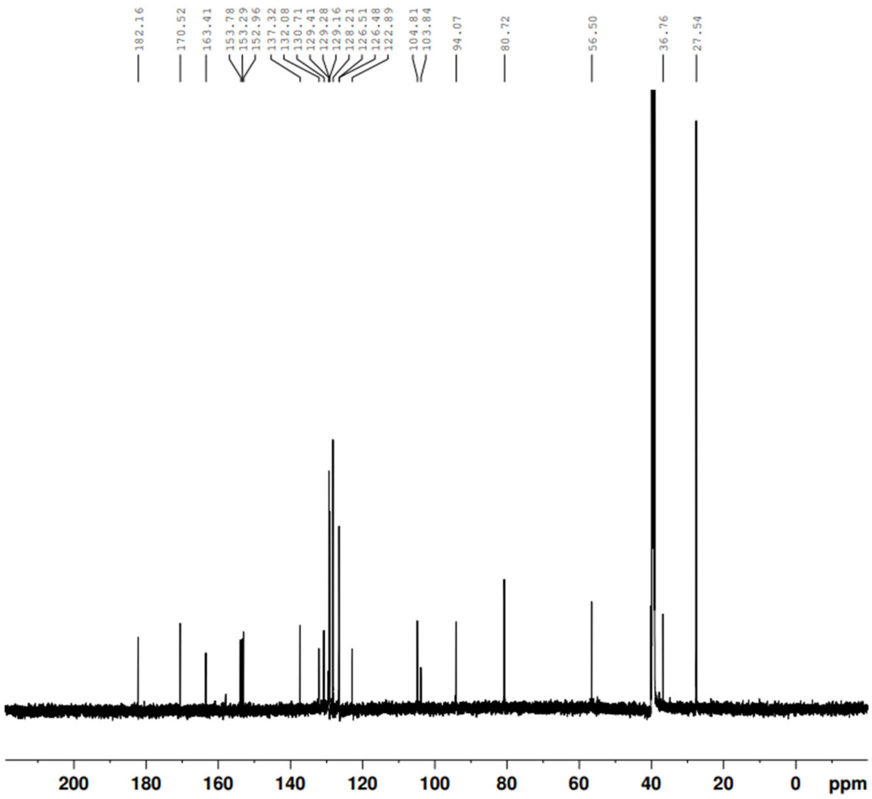

Figure S13. ^{13}C NMR spectra of compound **5c**.

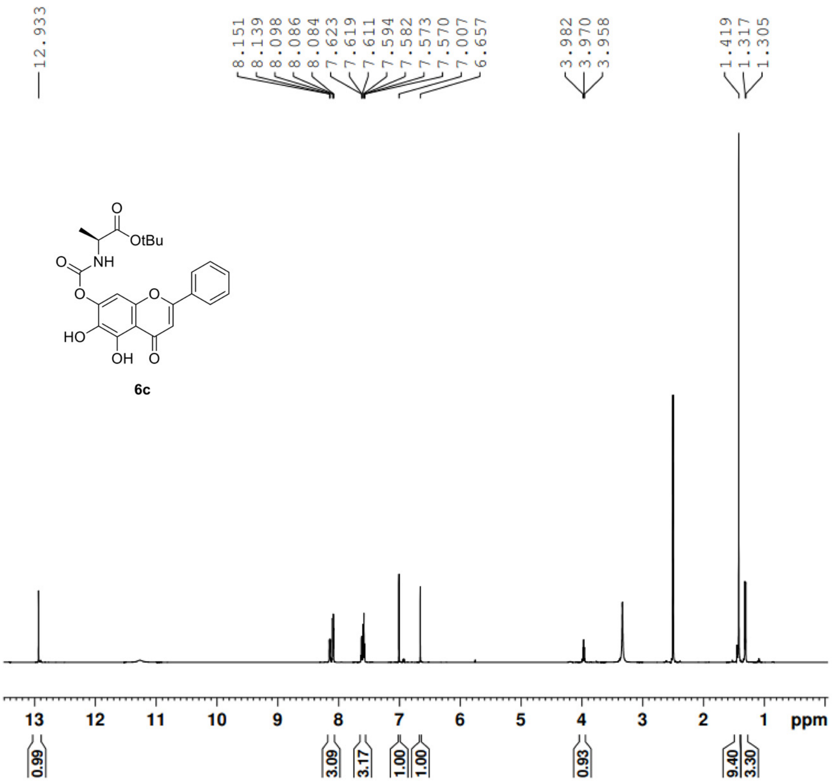

Figure S14. ^{1}H NMR spectra of compound **6c**.

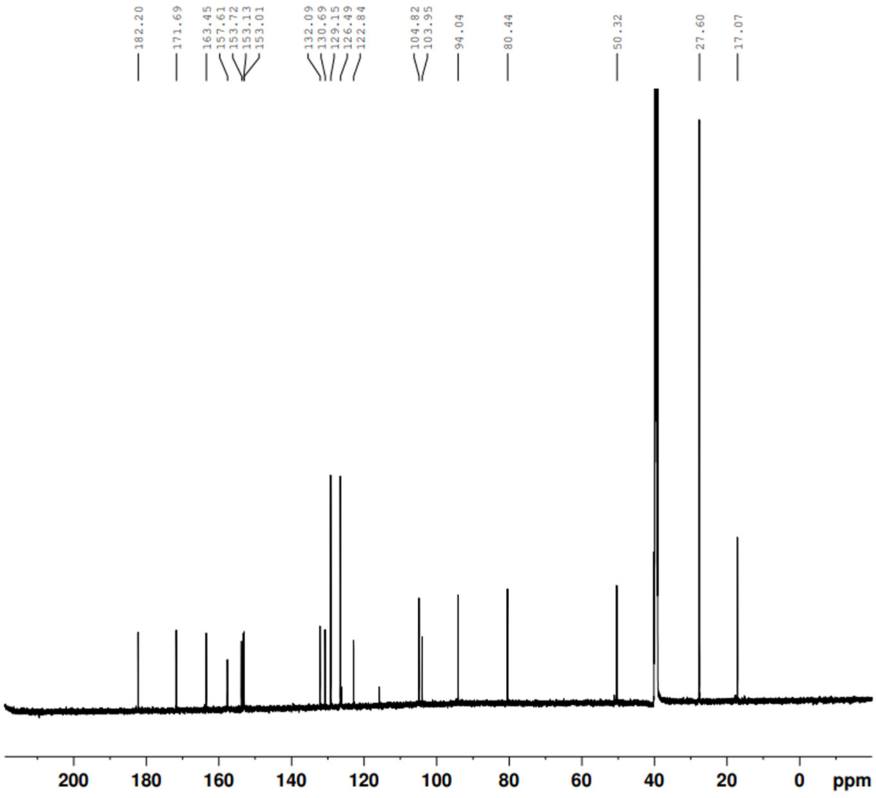

Figure S15. ^{13}C NMR spectra of compound **6c**.

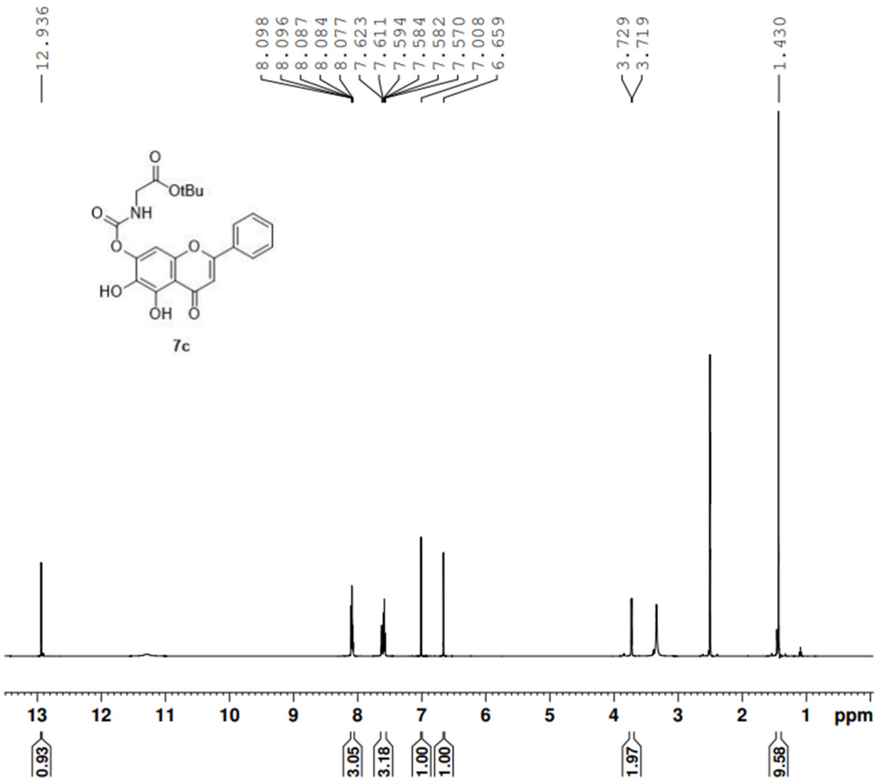

Figure S16. ^{1}H NMR spectra of compound **7c**.

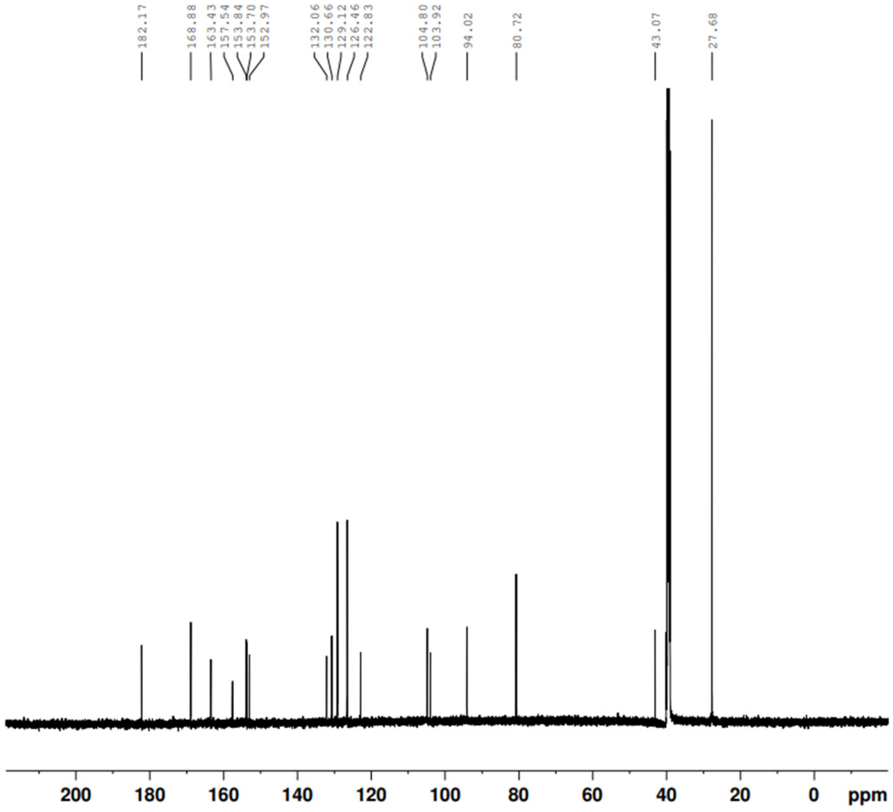

Figure S17. ^{13}C NMR spectra of compound **7c**.

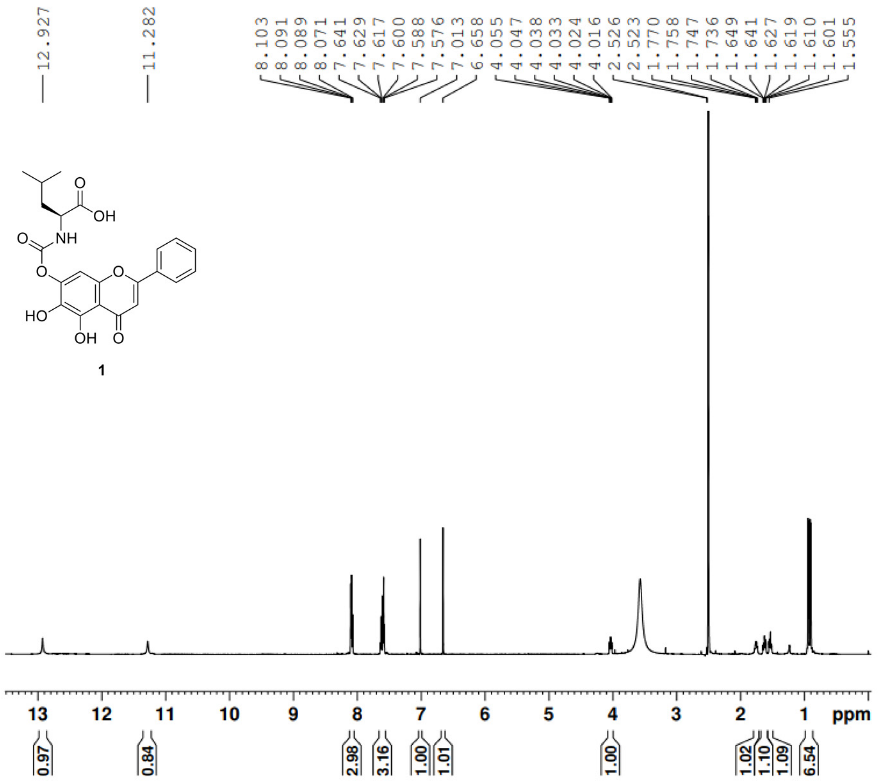

Figure S18. ^{1}H NMR spectra of compound **1**.

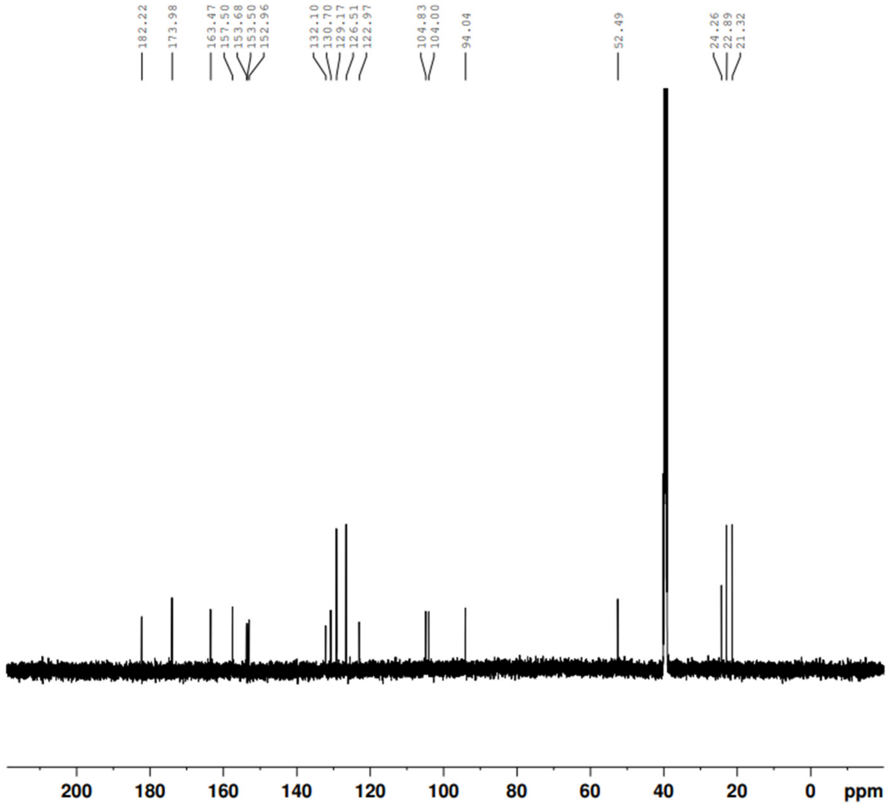

Figure S19. ^{13}C NMR spectra of compound **1**.

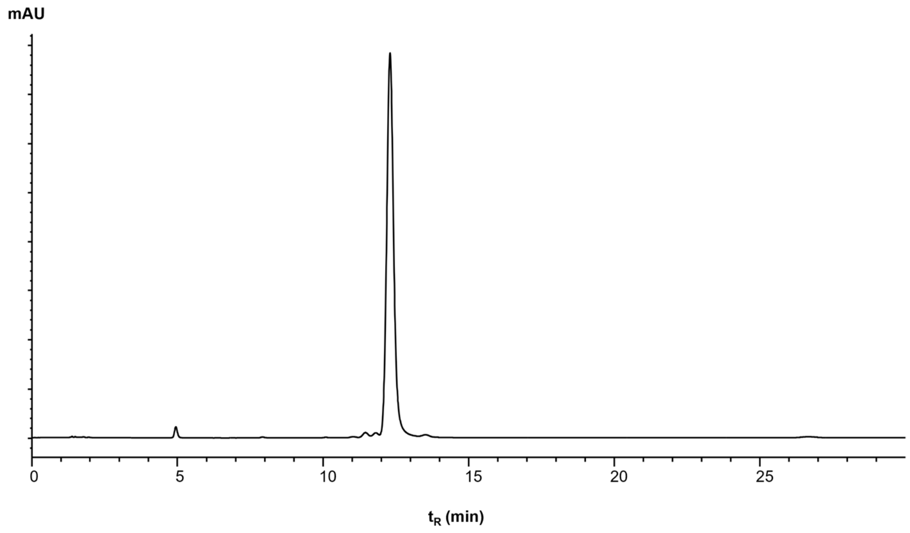

Figure S20. HPLC chromatogram of compound **1** eluted using Method C.

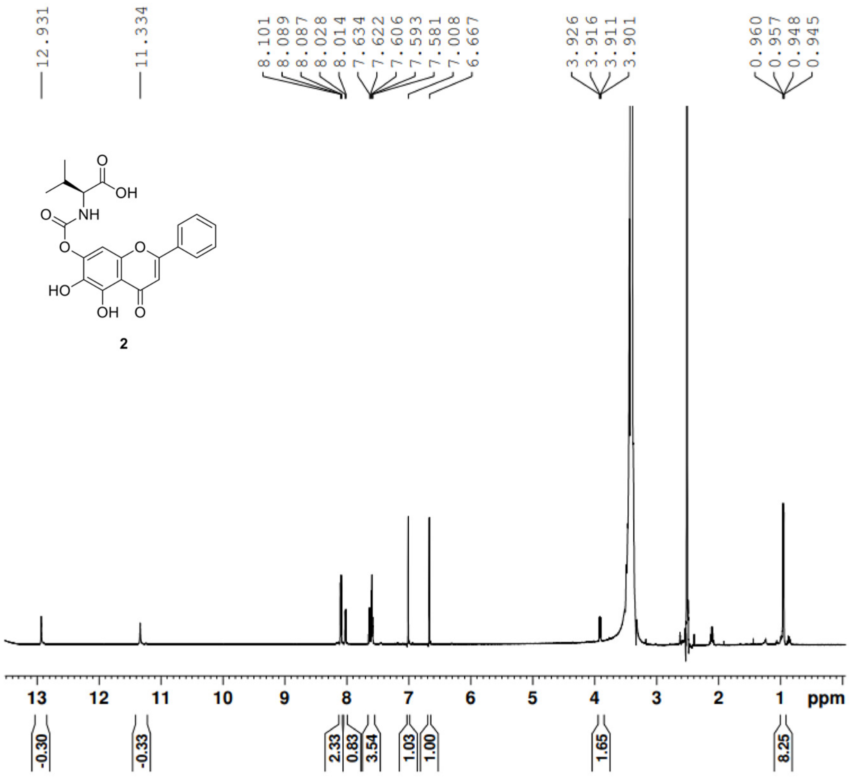

Figure S21. ^{1}H NMR spectra of compound **2**.

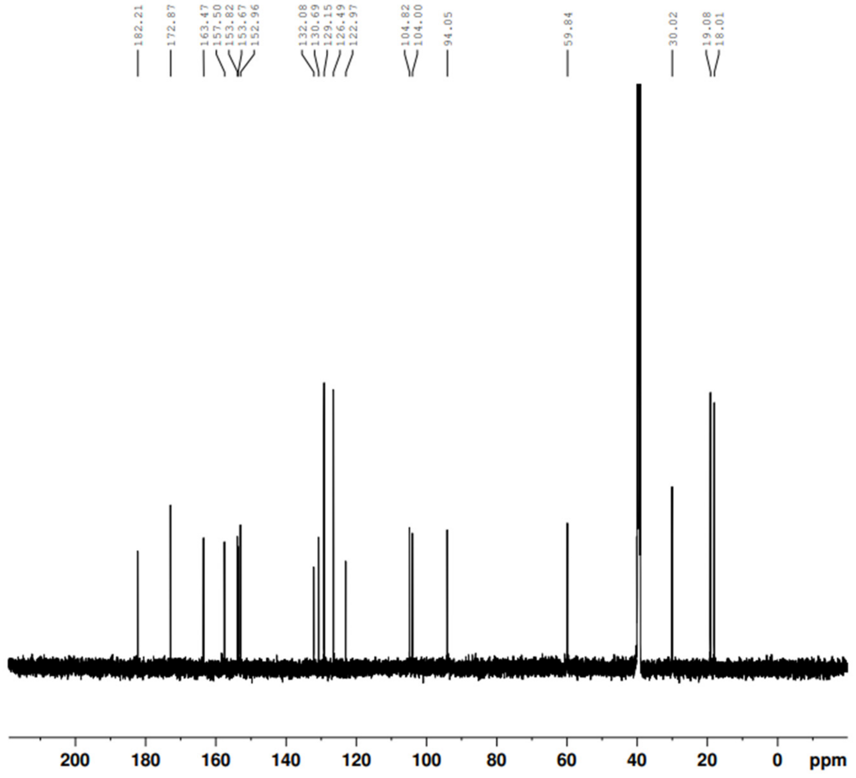

Figure S22. ^{13}C NMR spectra of compound **2**.

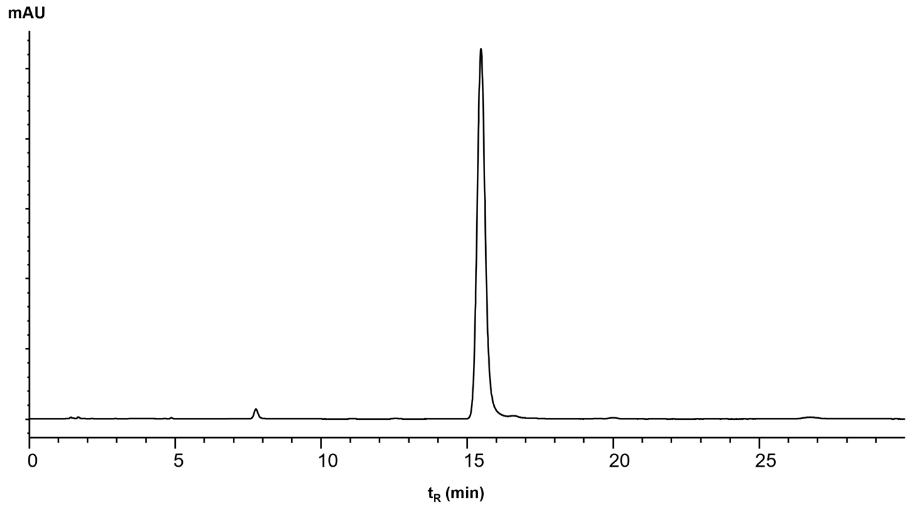

Figure S23. HPLC chromatogram of compound **2** eluted using Method C.

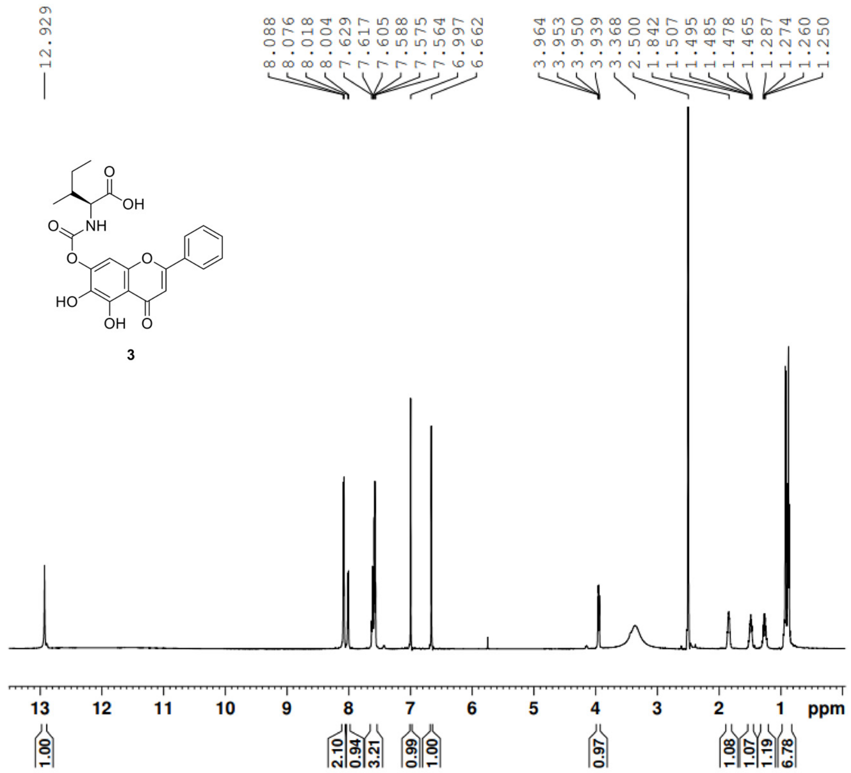

Figure S24. ^{1}H NMR spectra of compound **3.**

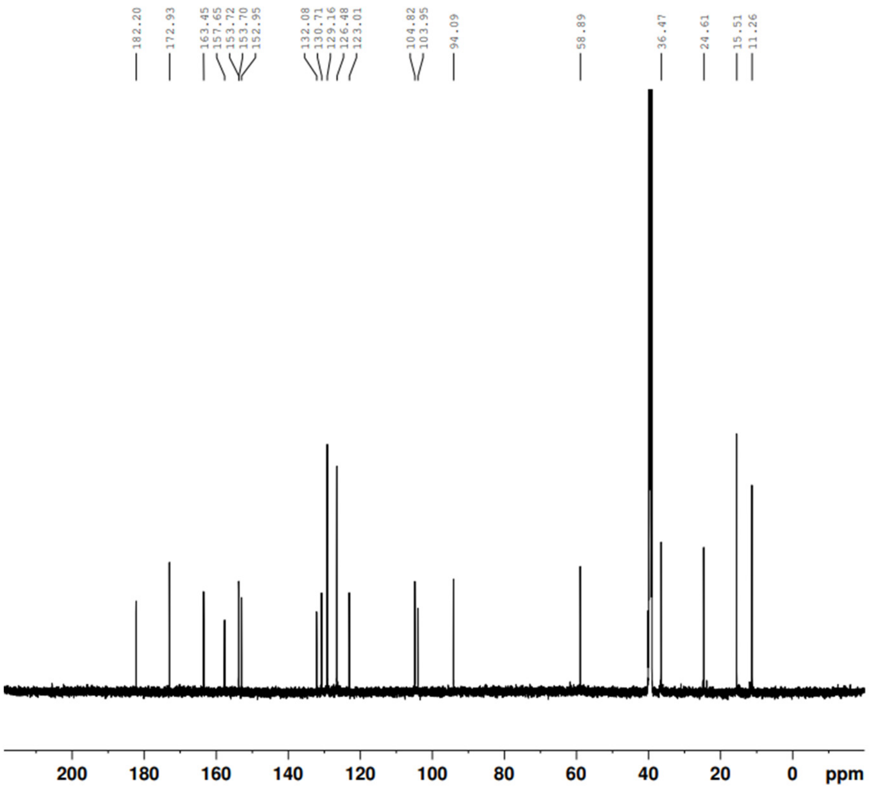

Figure S25. ^{13}C NMR spectra of compound **3**.

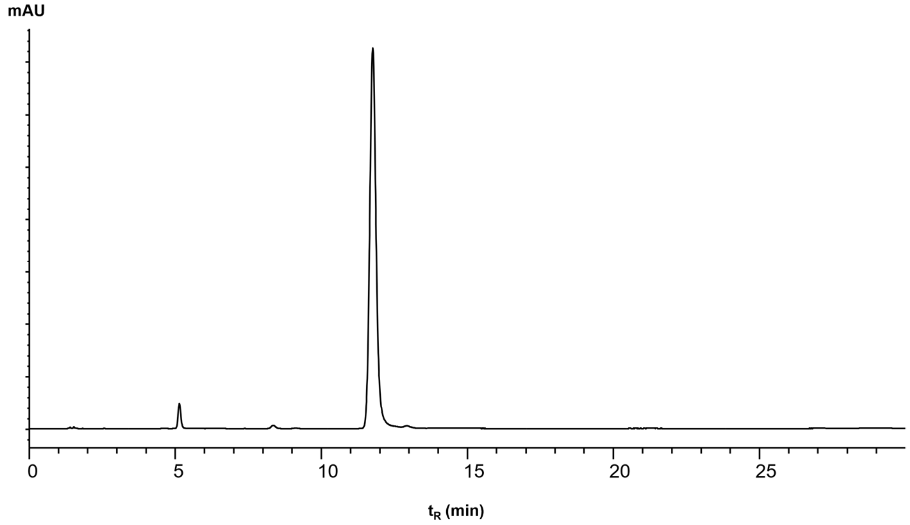

Figure S26. HPLC chromatogram of compound **3** eluted using Method C.

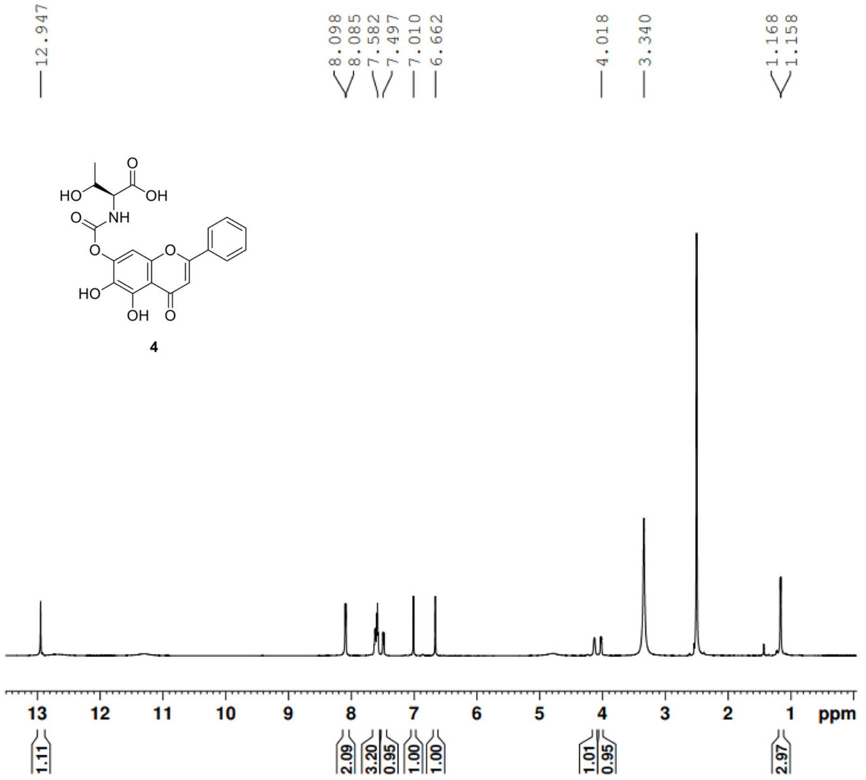

Figure S27. ^{1}H NMR spectra of compound **4**.

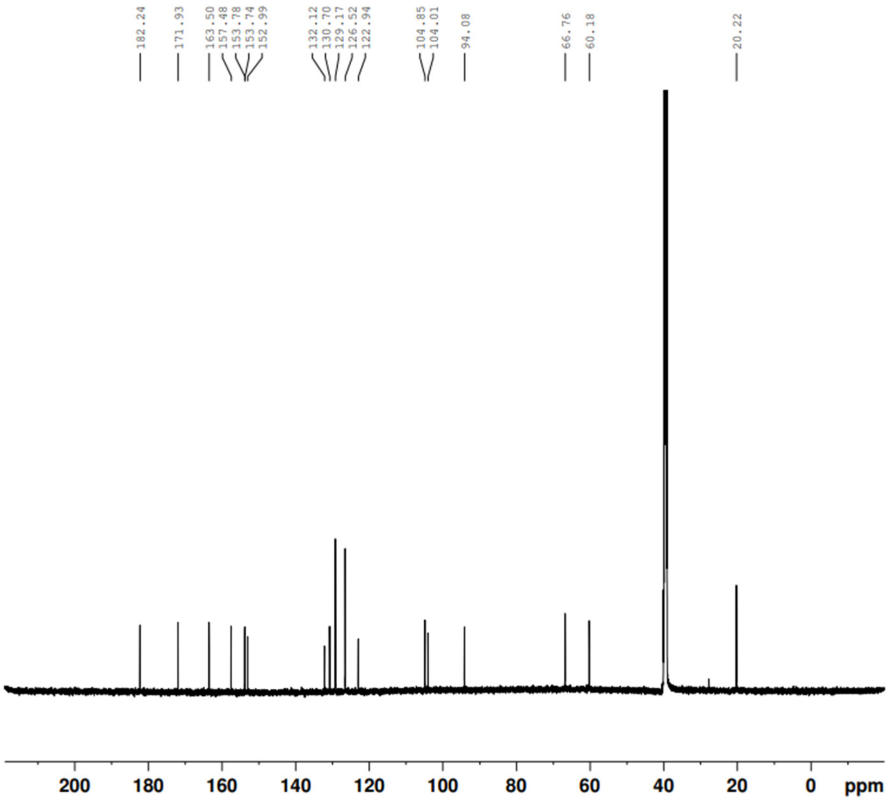

Figure S28. ^{13}C NMR spectra of compound **4.**

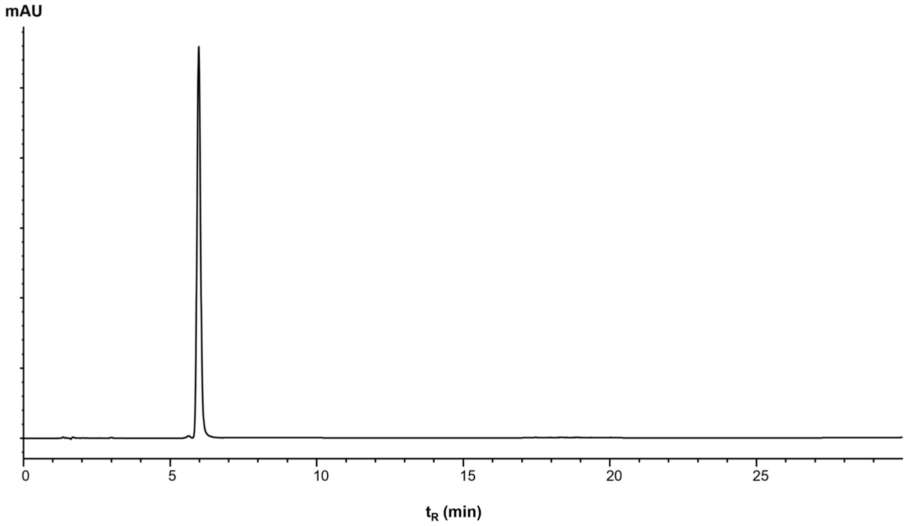

Figure S29. HPLC chromatogram of compound **4** eluted using Method B.

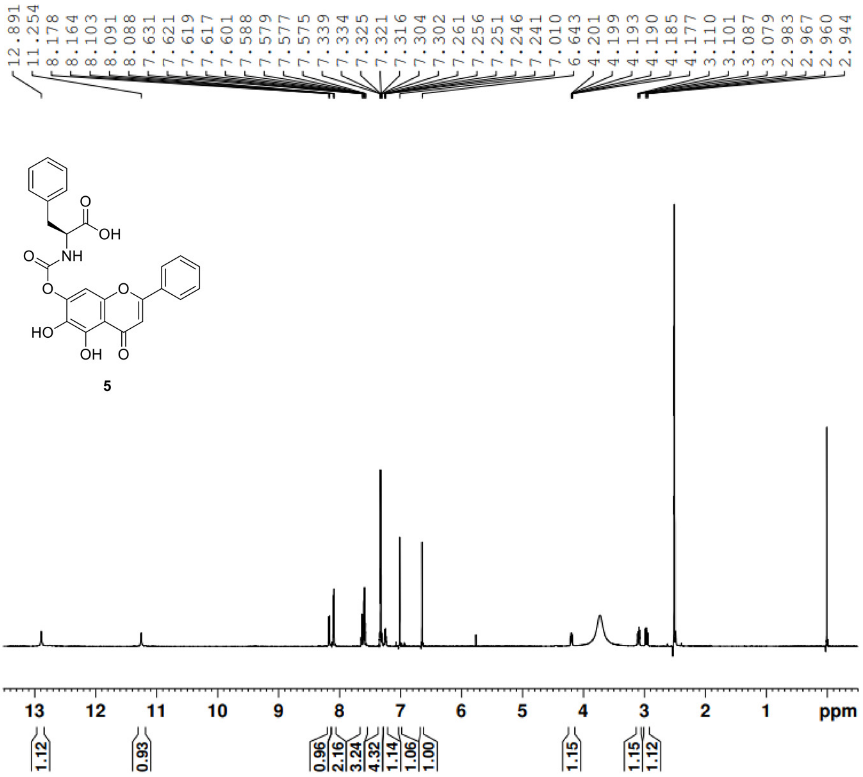

Figure S30. ^{1}H NMR spectra of compound **5.**

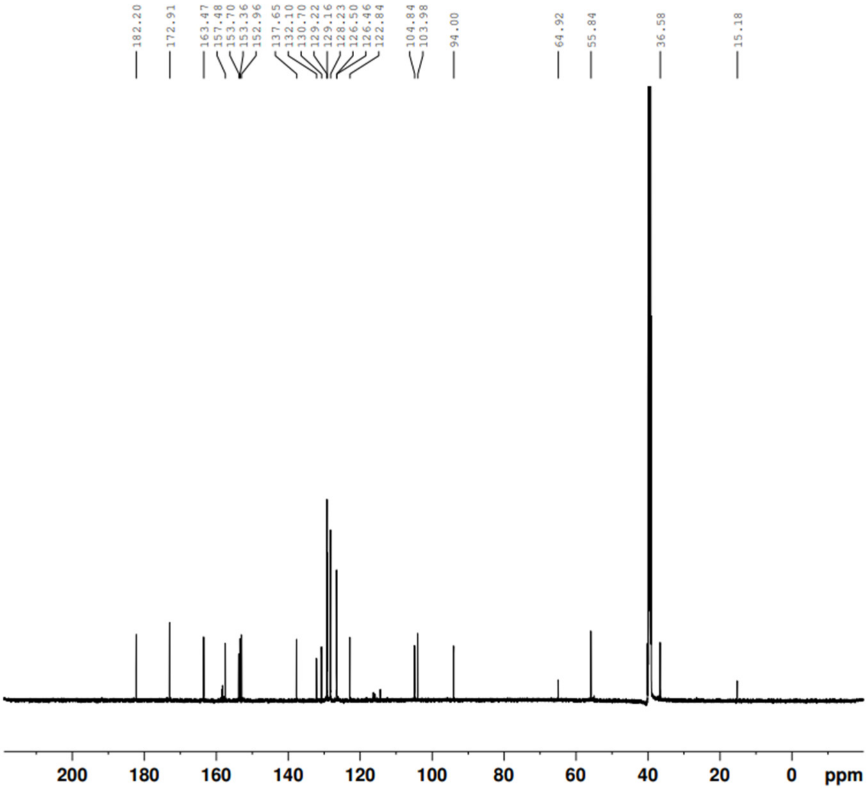

Figure S31. ^{13}C NMR spectra of compound **5**.

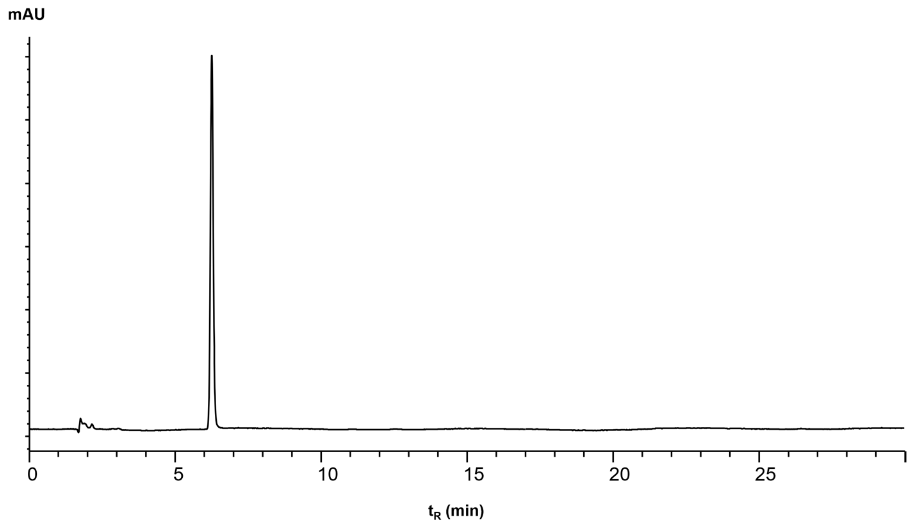

Figure S32. HPLC chromatogram of compound **5** eluted using Method A.

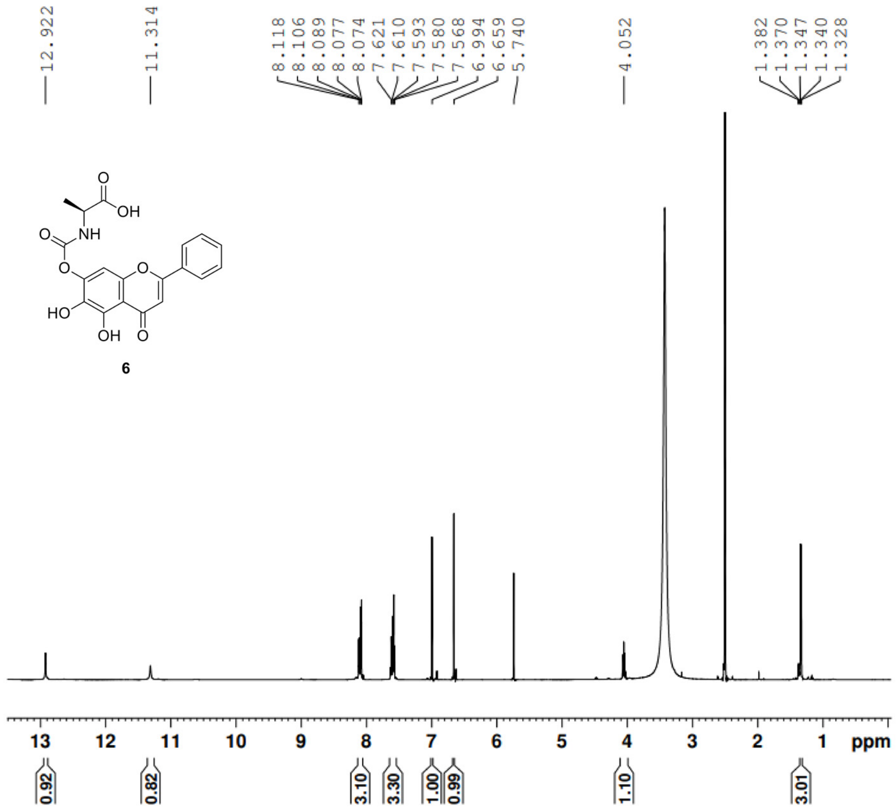

Figure S33. ^{1}H NMR spectra of compound **6**.

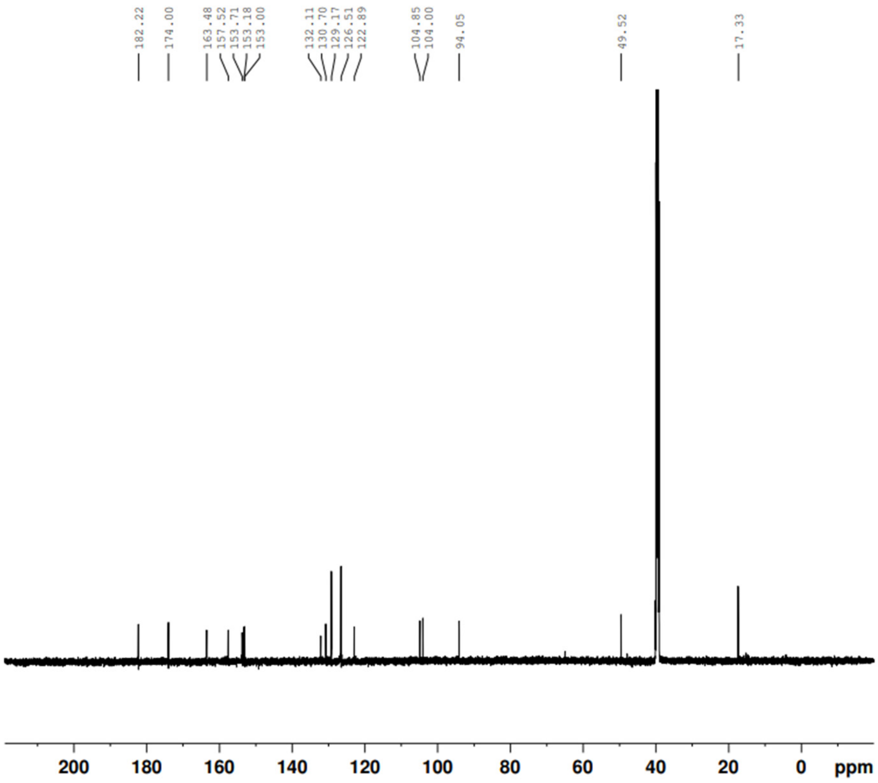

Figure S34. ^{13}C NMR spectra of compound **6**.

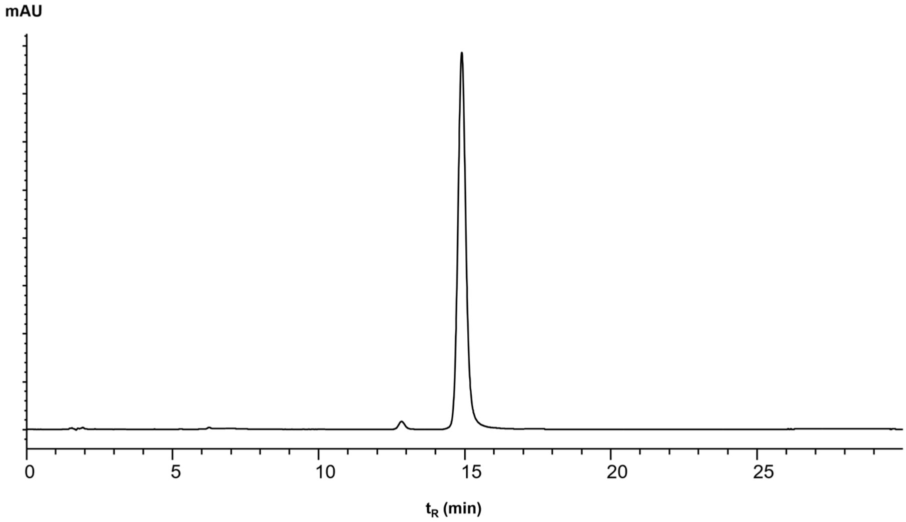

Figure S35. HPLC chromatogram of compound **6** eluted using Method A.

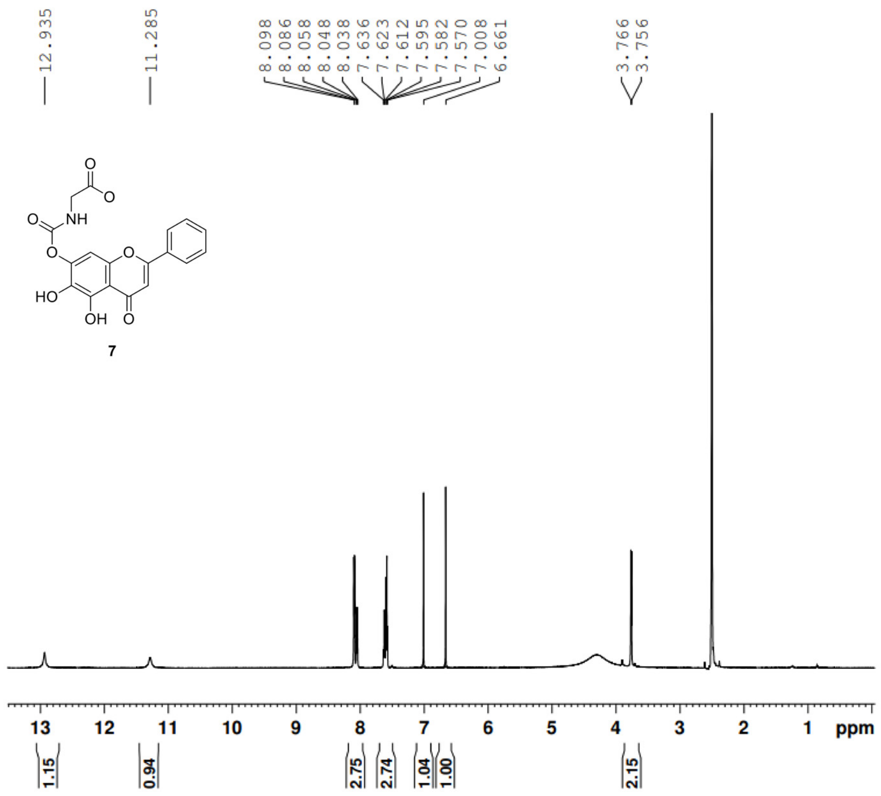

Figure S36. ^{1}H NMR spectra of compound **7**.

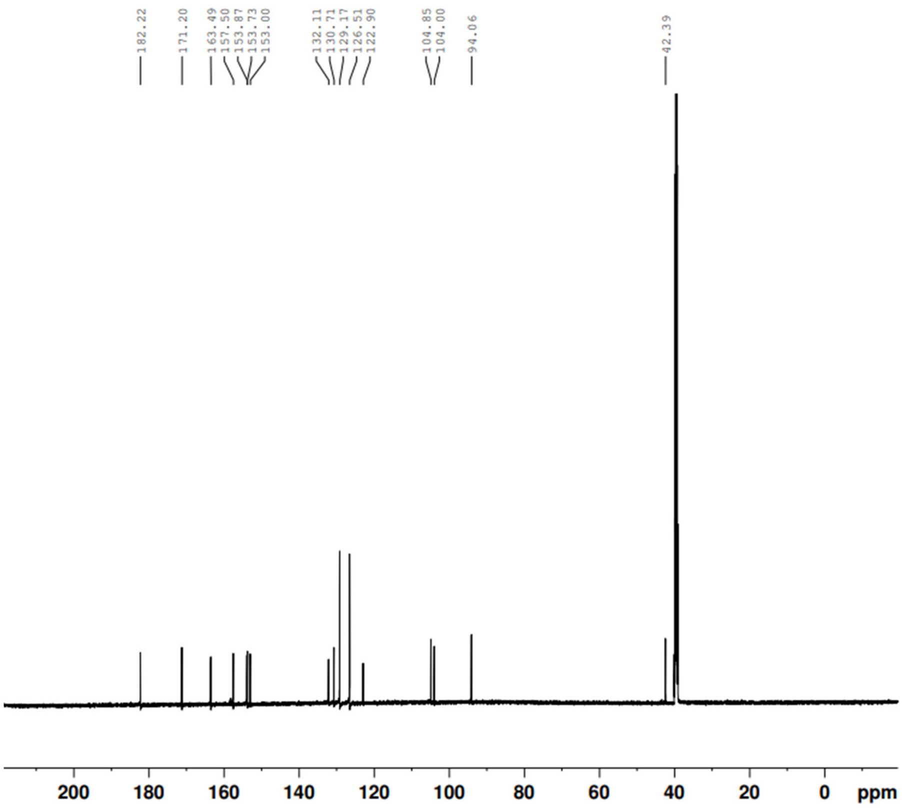

Figure S37. ^{13}C NMR spectra of compound **7**.

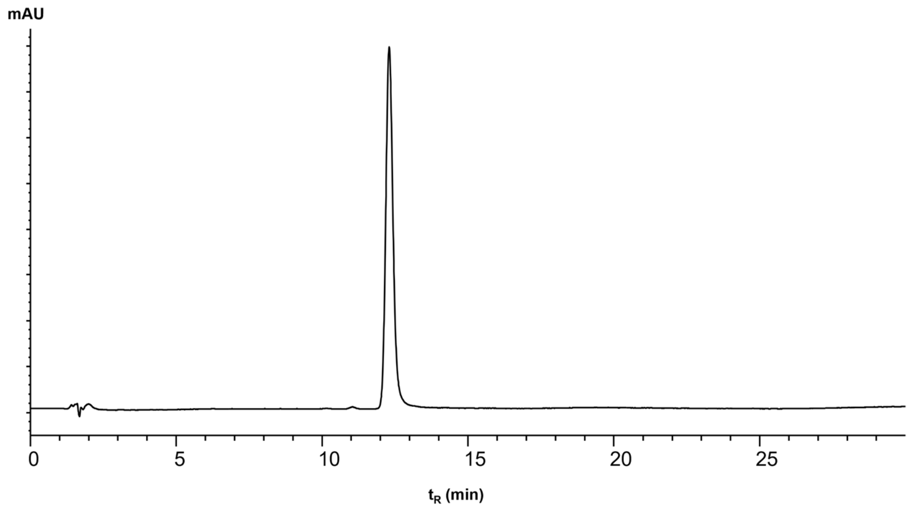

Figure S38. HPLC chromatogram of compound **7** eluted using Method A.
